# ANTXR1 deficiency promotes fibroblast senescence: implications for GAPO syndrome as a progeroid disorder

**DOI:** 10.1038/s41598-024-59901-y

**Published:** 2024-04-23

**Authors:** Matthias Przyklenk, Shreya Karmacharya, Debora Bonasera, Arthur-Lauri Pasanen-Zentz, Stanislav Kmoch, Mats Paulsson, Raimund Wagener, Gianmaria Liccardi, Alvise Schiavinato

**Affiliations:** 1 Center for Biochemistry, Medical Faculty, University of Cologne, Joseph-Stelzmann-Str. 52, 50931 Cologne, Germany; 2 Genetic Instability, Cell Death and Inflammation Laboratory, Center for Biochemistry, Medical Faculty, University of Cologne, Joseph-Stelzmann-Str. 52, 50931 Cologne, Germany; 3 Research Unit of Rare Diseases, Department of Pediatrics and Inherited Metabolic Disorders, First Faculty of Medicine, Charles University, Prague, Czech Republic; 4 Center for Molecular Medicine Cologne (CMMC), University of Cologne, Cologne, Germany; 5 Department of Pediatrics and Adolescent Medicine, Faculty of Medicine and University Hospital Cologne, University of Cologne, Cologne, Germany

**Keywords:** Clinical genetics, Medical genetics, Cytoskeleton, Mechanisms of disease, Senescence

## Abstract

ANTXR1 is one of two cell surface receptors mediating the uptake of the anthrax toxin into cells. Despite substantial research on its role in anthrax poisoning and a proposed function as a collagen receptor, ANTXR1’s physiological functions remain largely undefined. Pathogenic variants in ANTXR1 lead to the rare GAPO syndrome, named for its four primary features: Growth retardation, Alopecia, Pseudoanodontia, and Optic atrophy. The disease is also associated with a complex range of other phenotypes impacting the cardiovascular, skeletal, pulmonary and nervous systems. Aberrant accumulation of extracellular matrix components and fibrosis are considered to be crucial components in the pathogenesis of GAPO syndrome, contributing to the shortened life expectancy of affected individuals. Nonetheless, the specific mechanisms connecting ANTXR1 deficiency to the clinical manifestations of GAPO syndrome are largely unexplored. In this study, we present evidence that ANTXR1 deficiency initiates a senescent phenotype in human fibroblasts, correlating with defects in nuclear architecture and actin dynamics. We provide novel insights into ANTXR1's physiological functions and propose GAPO syndrome to be reconsidered as a progeroid disorder highlighting an unexpected role for an integrin-like extracellular matrix receptor in human aging.

## Introduction

ANTXR1, previously known as tumor endothelial marker 8 (TEM8), is a highly conserved integrin-like type I transmembrane protein that serves as the entry receptor for the anthrax toxin^1^ and the Seneca Valley virus^2^. Initially identified because of its upregulation in tumor endothelium^3^ murine Antxr1 was also found to be expressed in liver and brain endothelium during mouse embryonic development^4^. Studies using mice expressing LacZ under the control of the *Antxr1* promoter showed strong expression in blood vessels during embryonic development, and in other tissues such as the tailbud, cartilage primordia, vibrissae, and skin^5^. In chick embryos, ANTXR1 exhibits a dynamic expression in various tissues, including the craniofacial mesenchyme^6^. While ANTXR1 expression is generally low in adult tissues, it is believed to be upregulated in tumor-associated fibroblasts, endothelium, and pericytes^7,8^. Binding of the anthrax toxin protective antigen (PA) to the receptor is mediated by the metal ion-dependent adhesion site (MIDAS) within its single von Willebrand factor type A (VWA) domain, resembling an integrin-ligand interaction^9^. Receptor-bound PA is cleaved by a furin-type protease and forms oligomers that are endocytosed through clathrin and actin-dependent pathways^10^. While the role of ANTXR1 in anthrax poisoning is well-established, its physiological functions are still not fully understood. Like integrin VWA domains, the VWA domain of ANTXR1 has been found to mediate protein–protein interactions with the extracellular matrix (ECM), although there is uncertainty regarding the specific ECM components that bind to the receptor^8,11^. Additionally, ANTXR1 interacts with the cytoskeleton via its cytoplasmic tail, potentially by binding directly to actin^10,12^. Studies have demonstrated that the cytoplasmic tail and its interaction with the cytoskeleton can influence the conformation of ANTXR1, thereby modulating its binding affinity for PA^13,14^. Consequently, ANTXR1 connects the ECM and the cytoskeleton and may be endowed with inside-out signaling properties akin to integrins^15^. In addition, the interaction of ANTXR1 with the actin cytoskeleton was functionally confirmed by studies that uncovered a role of ANTXR1 in cell adhesion, spreading, and migration^16–18^. ANTXR1 has also been shown to form complexes with β1 integrin and to function as a sensor for extracellular mechanical cues^19–21^. Studies on Antxr1-deficient mice support the notion that ANTXR1 participates in regulating ECM homeostasis. These studies revealed focal tissue accumulation of certain ECM components, such as collagen I, collagen VI and fibronectin, although the underlying mechanisms leading to this abnormal ECM deposition remain under debate^5,22–24^. A significant insight into the role of ANTXR1 in tissue homeostasis arose from the discovery that pathogenic variants in ANTXR1 cause GAPO syndrome, a rare genetic disorder characterized by Growth retardation, Alopecia, Pseudoanodontia, and Optic atrophy^25^. GAPO syndrome reduces life expectancy, with affected individuals typically succumbing before the age of forty. In addition to the major features, GAPO patients experience a constellation of other signs and symptoms mainly involving connective tissues, including facial dysmorphisms, fibrosis, pulmonary hypertension, mental retardation, atherosclerosis, cardiomyopathy, hearing loss and various ocular defects^25–29^. However, the precise mechanisms by which loss of ANTXR1 leads to these severe phenotypes in affected individuals are not fully understood.

In this study, we investigated the effects of ANTXR1 deficiency in human fibroblasts. Interestingly, we observed that ANTXR1-deficient fibroblasts, and fibroblasts from GAPO syndrome patients, displayed several characteristics associated with cellular senescence. These features included the development of a senescence-associated secretory phenotype, reduced cell proliferation, and an expansion of the lysosomal compartment. Notably, these changes were also accompanied by morphological alterations in the nuclear architecture, which resemble those observed in fibroblasts from individuals with Hutchinson-Gilford progeria^30^. Given the known functions of ANTXR1 in cell–matrix adhesion, we hypothesized a link between the nuclear alterations and disrupted cytoskeleton dynamics, implicating a specific role for the actin nucleation Arp2/3 complex. In summary, our findings emphasize the importance of ANTXR1 in preventing cellular senescence in fibroblasts and suggest that this role may explain the observed phenotypic similarities between GAPO patients and those affected with other progeroid disorders^31^.

## Results

### ANTXR1-deficient fibroblasts exhibit hallmarks of cellular senescence and changes in nuclear architecture

**Figure 1:**
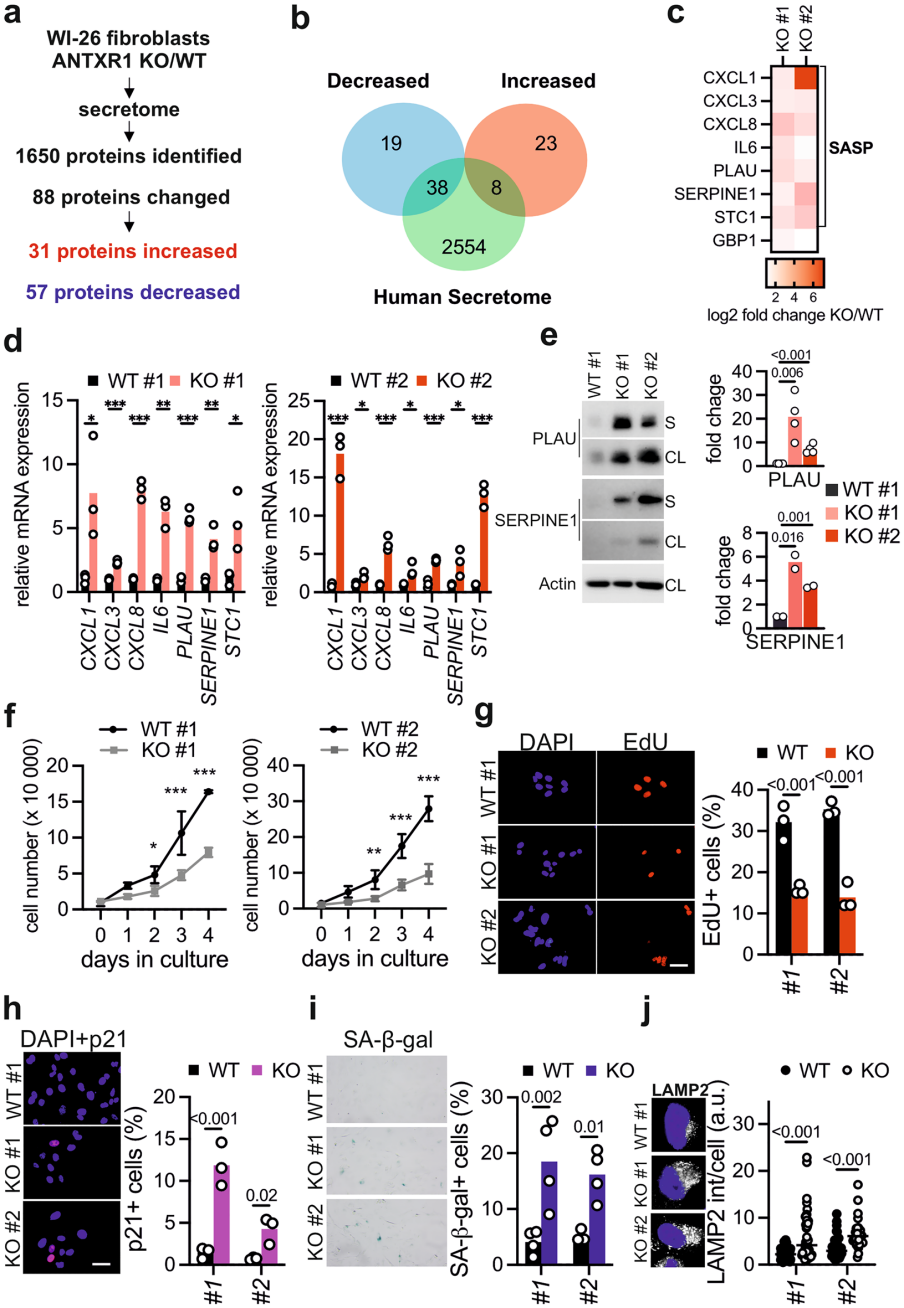
ANTXR1-deficient fibroblasts show hallmarks of cellular senescence (**a**) Schematic overview of the experimental workflow for the secretome analysis. Control and ANTXR1-deficient WI-26 fibroblasts at confluency were kept in serum free medium supplied with ascorbate. After 4 days the media were collected, digested and subjected to label-free quantitative mass spectrometric analysis. Shown data represent the combined results from two independent experiments from two different control and knockout fibroblast clones. Triplicates were analyzed for each cell line. (**b**) Venn diagram of proteins significantly decreased and increased and their distribution within the human secretome dataset. (**c**) Heatmap showing the abundance of the eight secreted proteins whose secretion was found to be significantly increased in the media of both ANTXR1-deficient clones. The fold change of the two knockout clones was compared to their relative controls. A bracket indicates the seven proteins that are known components of the Senescence-Associated Secretory Phenotype (SASP). (**d**) qPCR of the seven genes coding for the SASP components identified in the secretome analysis. Unpaired t-test. *p < 0.05, **p < 0.01, ***p < 0.001. (**e**) Cropped PLAU and SERPINE1 immunoblots of cell lysates (CL) and serum-free supernatants (S) of control and ANTXR1-deficient WI-26 cells. Graphs show the quantification of the two proteins in the conditioned media relative to the actin intensity of the cell lysate. Unpaired t-test. Original uncropped immunoblots are presented in the Supplementary Information file. (**f**) Growth curves of control and ANTXR1-deficient WI-26 clones. 10.000 cells were plated and maintained in FCS-containing medium for the indicated number of days. For each time point, cell numbers of triplicate samples were counted twice and the average was used. Two-way ANOVA, *p < 0.05, **p < 0.01, ***p < 0.001. (**g**) EdU incorporation in WI-26 control and ANTXR1-deficient cells. Representative images and quantification (n = 3). Each dot represents the average of at least five microscopic fields for each sample. At least 100 cells were analyzed for each cell line. (**h**) Representative images and quantification of p21 positive nuclei (magenta) in control and ANTXR1-deficient WI-26 clones (n = 3). Each dot represents the average of at least five microscopic fields for each sample. At least 100 cells were analyzed for each cell line. (**i**) Representative images and quantification of senescence-associated β-galactosidase (SA-β-gal) positive control and ANTXR1-deficient WI-26 cells (n = 4). SA-β-gal positive cells from five microscopic fields were counted for each sample. (**j**) Representative images and quantification of LAMP2 staining in control and ANTXR1-deficient WI-26 cells. For the quantification, the fluorescence intensity of at least 30 cells per cell type was measured. Two-way ANOVA (**g**–**j**).

**Figure 2:**
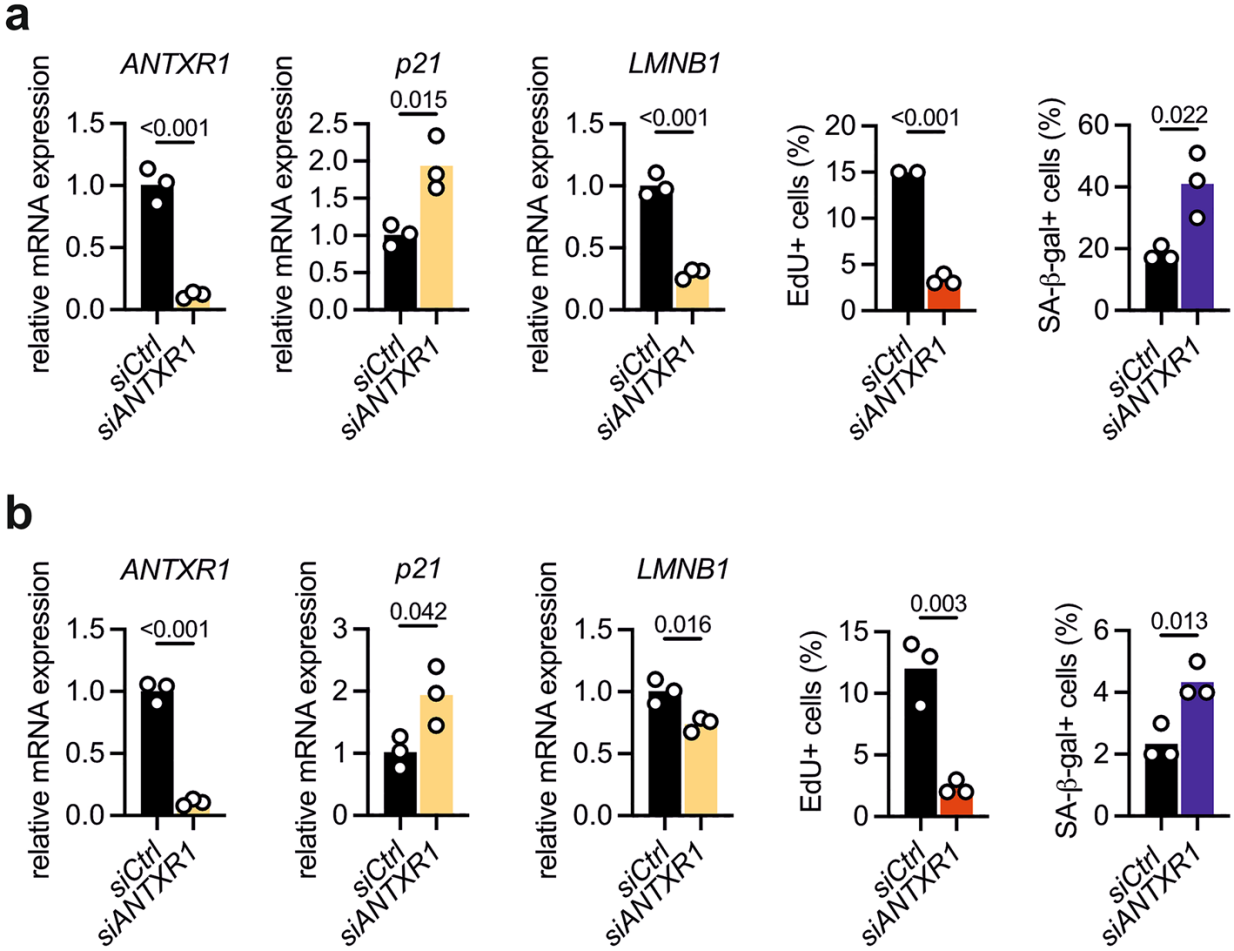
ANTXR1 knockdown induces senescence features in primary fibroblasts. Primary human dermal fibroblasts of 8-year-old (**a**) and 20-year-old (**b**) donors were reverse transfected with a control siRNA or with a pair of siRNAs targeting *ANTXR1*. Four days after transfection, RNA was isolated and *ANTXR1*, *p21* and *LMNB1* expression quantified by qPCR. Unpaired t-test. At the same time point EdU incorporation and SA-β-Gal expression were analyzed. > 100 cells were analyzed for each sample. n = 3, unpaired t-tests.

Previous studies in fibroblasts have proposed a cell-autonomous role of ANTXR1 in regulating the composition of the extracellular environment^5,24^. To further investigate this, we used CRISPR/Cas to generate ANTXR1-deficiency in WI-26 cells, an SV40-immortalized embryonic human lung fibroblast cell line. We independently generated two control and two knockout clones that were validated by western blot, qPCR (Supplementary Fig. 1), and DNA sequencing (not shown). To assess how ANTXR1 deficiency affects the extracellular environment, we performed label-free quantitative proteomics on conditioned media of two control and two ANTXR1-deficient fibroblast clones. Our analysis identified 1650 proteins, of which 88 showed altered levels in ANTXR1-deficient cells compared to the controls (log2 fold change ≥ 1, p-value < 0.05). Specifically, 31 proteins were significantly upregulated, while 57 proteins were downregulated in both ANTXR1-deficient clones (Fig. 1a, Supplementary Fig. 2a and Supplementary Table 1). To focus on secreted proteins, we intersected our list of upregulated and downregulated proteins with a list encompassing the whole human secretome^32^ and found that 38 secreted proteins were downregulated in ANTXR1-deficient cells, while 8 were upregulated (Fig. 1b). Gene ontology analysis revealed an enrichment of extracellular matrix-related terms for the downregulated secreted proteins, while the upregulated proteins were associated with inflammation-related terms (Supplementary Fig. 2b). Notably, 7 out of the 8 upregulated proteins were known components of the Senescence-Associated Secretory Phenotype (SASP), a characteristic feature of cellular senescence^33,34^ (Fig. 1c and Supplementary Fig. 2c). We validated the increased transcription levels of all identified SASP components through qPCR (Fig. 1d). Additionally, we confirmed the increased protein levels of two components, PLAU and SERPINE1, by western blot (Fig. 1e) and casein zymography (Supplementary Fig. 3). The observation that loss of ANTXR1 leads to a shift toward the SASP prompted us to investigate other characteristics of cellular senescence in ANTXR1-deficient WI-26 fibroblasts. We therefore examined their proliferative capacity and found that ANTXR1-deficient cells display reduced proliferation rates and decreased incorporation of the thymidine analogue EdU (Fig. 1f and g). Activation of the p53/p21 pathway is a common mechanism underlying proliferation inhibition in senescent cells^35^. Consistently, ANTXR1-deficient WI-26 cells exhibited an increased proportion of p21-positive nuclei, as revealed by immunostaining (Fig. 1h). Moreover, ANTXR1-deficient WI-26 fibroblasts demonstrated elevated levels of lysosomal senescence-associated β-galactosidase (SA-β-gal) activity (Fig. 1i). The lack of ANTXR1 was also associated with an increased lysosomal mass, as indicated by enhanced LAMP2 staining intensity (Fig. 1j). Altered extracellular matrix biosynthesis is another hallmark of senescent cells, particularly in fibroblasts^33,36,37^, and abnormalities in ECM homeostasis have been reported for GAPO patients and Antxr1-deficient mice. Our unbiased mass spectrometry analysis revealed a general reduction in the secretion of ECM components by ANTXR1-deficient WI-26 fibroblasts (Fig. 1a,b and Supplementary Fig. 2). Consequently, we conducted further investigations into the expression, secretion, and network formation of three major ECM components: collagen I, collagen VI, and fibronectin. These components have been associated with ANTXR1 deficiency in previous studies^38–40^ and consistently, we observed significant decreased levels in our secretome analysis (Supplementary Table 1). ANTXR1-deficient WI-26 cells exhibited altered synthesis of these three ECM components, as confirmed by qPCR, western blot, and immunofluorescence staining (Supplementary Fig. 4), supporting the robustness of our findings. Next, we aimed to investigate whether ANTXR1 deficiency could induce a senescent phenotype in non-transformed primary dermal fibroblasts. To achieve this, we performed reverse transfection with ANTXR1-specific siRNAs^41^ or a control siRNA in dermal fibroblasts derived from 8 and 20 year-old healthy donors. Analysis by qPCR demonstrated a significant reduction in *ANTXR1* mRNA levels up to four days post-transfection. Concurrently, ANTXR1-depleted fibroblasts exhibited increased expression of *p21*, decreased *LMNB1* expression and decreased EdU incorporation. Furthermore, a larger proportion of ANTXR1-depleted fibroblasts displayed higher SA-β-Gal positivity compared to control siRNA-transfected cells (Fig. 2). Similar results were obtained with dermal fibroblasts derived from a 53-year-old donor (Supplementary Fig. 5). In contrast to our observations in WI-26 cells, ANTXR1 depletion did not induce a SASP in these primary dermal fibroblasts. (Supplementary Fig. 5a–f). We attributed this to the age of the donor and therefore carried out the same experiments with primary fibroblasts derived from a much older donor (96-year-old). Interestingly, knockdown of *ANTXR1* in these primary dermal fibroblasts showed increased expression levels of *p21* and SASP components *IL6* and *CXCL8*, suggesting that other age-dependent factors sensitize the cells to SASP induction upon ANTXR1 deficiency in primary fibroblasts (Supplementary Fig. 5g–j). Overall, these results indicate that ANTXR1-deficient fibroblasts acquire a phenotype that is consistent with cellular senescence.

### GAPO patient fibroblasts exhibit hallmarks of cellular senescence and changes in nuclear architecture

**Figure 3:**
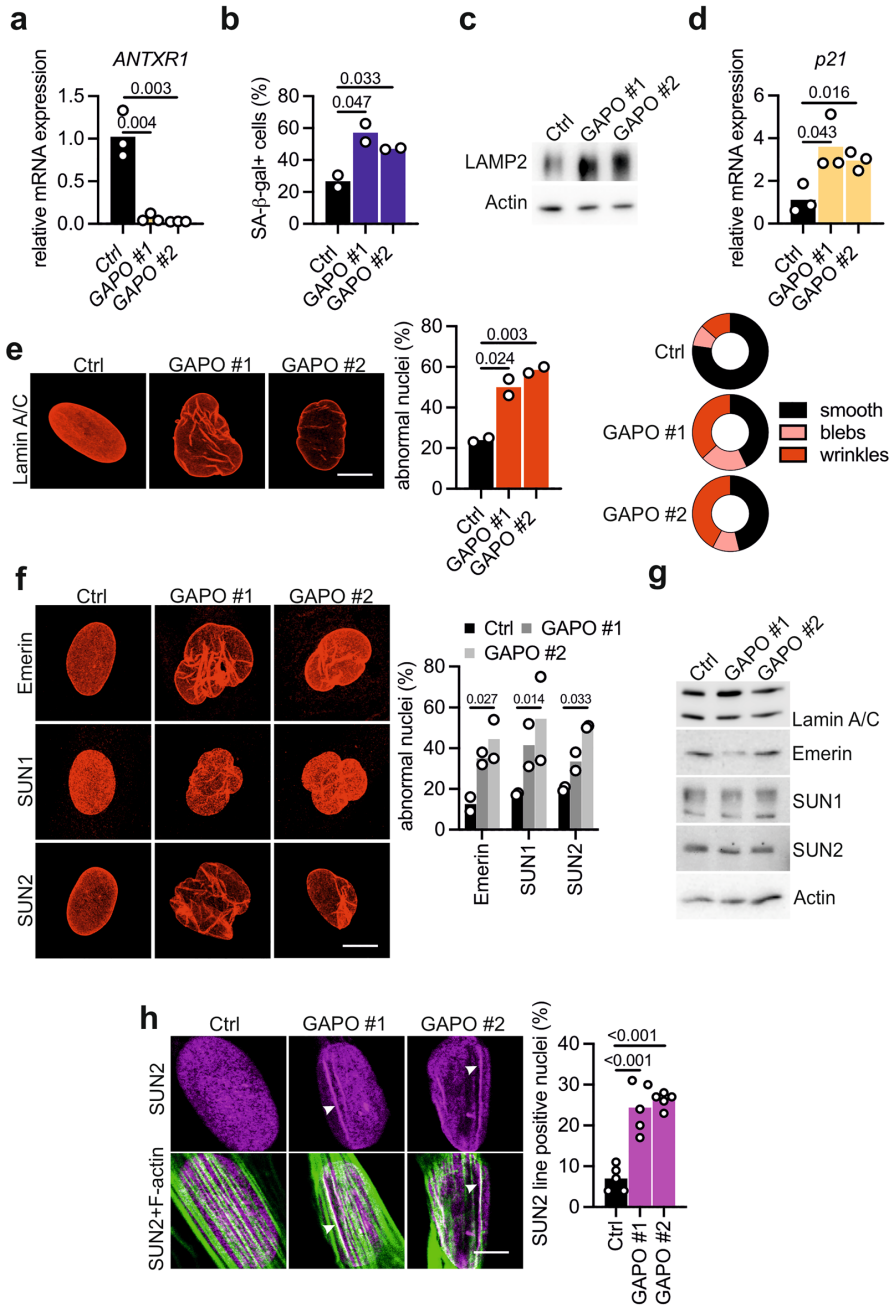
Fibroblasts of GAPO patients exhibit hallmarks of cellular senescence and an altered nuclear architecture. (**a**) qPCR analysis of *ANTXR1* expression in primary dermal fibroblasts derived from two GAPO patients carrying homozygous ANTXR1 p.Arg169* (GAPO #1) and p.Arg88* (GAPO #2) substitutions and a healthy donor (Ctrl). (**b**) Increased SA-β-gal levels in the fibroblasts of GAPO patients (n = 2, > 100 cells per sample). Unpaired t-test. (**c**) Western blot analysis (cropped) for LAMP2 revealed an increased lysosomal mass in GAPO fibroblasts. (**d**) *p21* mRNA expression levels in the indicated fibroblasts as assessed by qPCR. (**e**,**f**) Nuclear envelope defects in fibroblasts of GAPO patients as revealed by confocal images and quantified by (**e**) lamin A/C and (**f**) emerin, SUN1 and SUN2 immunostaining (n = 2). At least 100 cells per sample were counted. Unpaired t-test. Scale bars: 10 µm (**g**) Representative cropped immunoblots for the indicated nuclear envelope proteins in control and GAPO patient fibroblasts. Actin was used as a loading control. (**h**) Representative confocal immunofluorescence images of nuclei of control and GAPO fibroblasts stained for the LINC complex component SUN2 (magenta) and with 488-conjugated phalloidin (green) to visualize actin filaments. Note the presence of SUN2-positive nuclear lines in GAPO fibroblasts that overlap with dorsal actin fibers (arrowheads). Scale bar: 10 µm. Quantification of SUN2-positive nuclear lines in the indicated fibroblasts grown for 24 h on glass coverslips is displayed on the right. At least 100 cells per sample were counted. Original uncropped immunoblots are presented in Supplementary Information file .

To further validate our findings, we analyzed primary dermal fibroblasts obtained from two GAPO patients harboring homozygous nonsense pathogenic variants in *ANTXR1*^25^. qPCR analysis confirmed the absence of ANTXR1 expression in these cells (Fig. 3a). It is noteworthy that the healthy control fibroblasts were obtained from a donor 53-year-old at the time of biopsy, while the two GAPO patients were 18 (GAPO #1) and 10 years old (GAPO #2). Nevertheless, GAPO fibroblasts exhibited increased lysosomal mass as indicated by higher levels of SA-β-gal and LAMP2, and slightly increased *p21* expression (Fig. 3b–d). Similar to siRNA-mediated ANTXR1 depletion in primary fibroblasts from the 53-year-old donor, qPCR analysis of SASP components did not reveal increased expression in GAPO patient fibroblasts compared to control cells (not shown and Supplementary Fig. 10). Cellular senescence is a hallmark of aging^42^ and accumulation of senescent cells within tissues has been linked to the ageing process^43,44^. Notably, patients with GAPO syndrome display phenotypic features, including growth retardation, alopecia, dental anomalies and characteristic facial dysmorphisms, similar to individuals with progeroid disorders. These rare genetic conditions are characterized by premature aging and are predominantly caused by mutations in genes involved in maintaining genome integrity^31^. Hutchinson-Gilford Progeria Syndrome (HGPS) is the most well-known premature aging disorder. It is caused by mutations in the *LMNA* gene, which encodes lamin A and lamin C, intermediate filament proteins of the nuclear lamina^45^. In vitro, HGPS fibroblasts progressively accumulate a mutant form of lamin A, leading to nuclear structural defects^30^. We hypothesized that fibroblasts lacking ANTXR1 might also exhibit nuclear envelope defects. To investigate this, we stained nuclei for lamin A/C and observed a range of nuclear envelope alterations, including wrinkling of the lamina and deformation characterized by blebbing and invaginations. These defects were significantly more frequent in primary fibroblasts of GAPO patients than in control cells (Fig. 3e). Similar results were obtained also via siRNA mediated-ANTXR1 knockdown in primary dermal fibroblast (not shown). Immunostaining for lamin A/C revealed a higher abundance of nuclear envelope defects also in ANTXR1-deficient WI-26 fibroblasts compared to control cells (Supplementary Fig. 6a,b). Nuclear envelope abnormalities have been associated with DNA damage, which subsequently activates NF-κB, playing a critical role in SASP induction. To confirm the concurrent activation of NF-κB in our system, we examined the phosphorylation status of p65 and IKKα/β. These proteins not only serve as indicators of pathway activation but are also specifically linked to the induction of the senescence-associated proinflammatory phenotype^46,47^. Consistently, immunoblotting using phospho-specific antibodies for p65 and IKKα/β revealed higher phosphorylation levels in ANTXR1-deficient WI-26 cells compared to control cells (Supplementary Fig. 6c,d). Furthermore, inhibition of IKK activity significantly reduced the upregulation of *IL6* and *CXCL8* in ANTXR1-deficient cells (Supplementary Fig. 6e), suggesting that the SASP is a consequence of intrinsic NF-κB activation in these cells.

### ANXTR1 deficiency leads to nuclear envelope defects through altered actin dynamics

**Figure 4:**
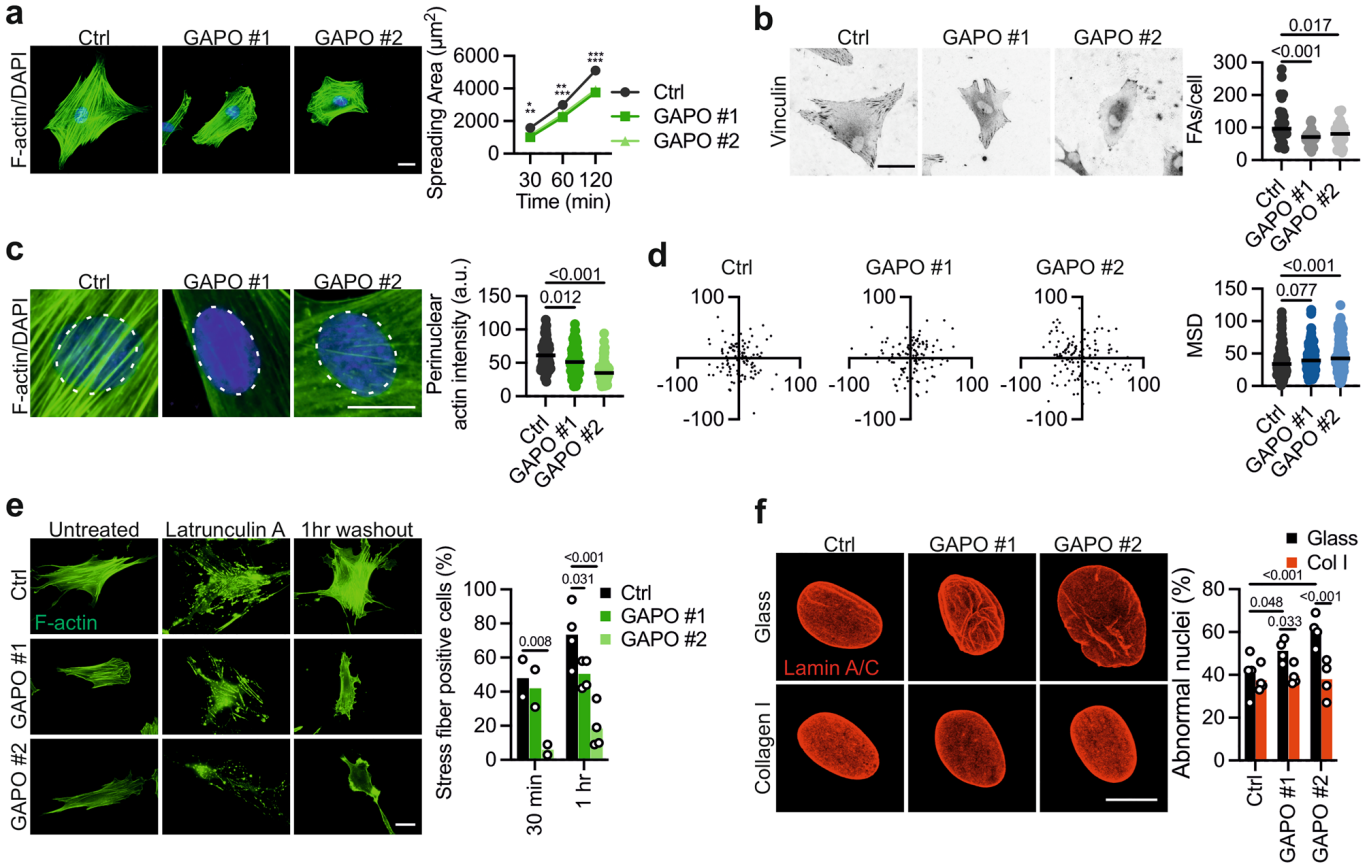
Altered actin dynamics in GAPO fibroblasts. (**a**) Representative images of control and GAPO fibroblasts stained for filamentous actin with phalloidin (green) after spreading for 2 h on collagen I. Scale bar = 10 µm. The graph shows quantification of the spreading area of control and GAPO fibroblasts on collagen I after the indicated intervals. Data are combined from three independent experiments, with over 100 cells measured per time point for each cell line in each experiment. Two-way ANOVA, *p < 0.05, **p < 0.01, ***p < 0.001. (**b**) Representative images of control and GAPO fibroblasts stained for vinculin to visualize the focal adhesions. Quantification of the number of focal adhesions per cell in the indicated fibroblast lines is shown on the right. Data are merged from two independent experiments, with at least 50 cells analyzed for each group. Unpaired t-test. (**c**) Example of F-actin staining (green) in the perinuclear area of the indicated fibroblasts. Nuclei are counterstained with DAPI (blue). The white dotted circles represent the regions that were used for the quantification of the fluorescence intensity that is shown on the right. Data are merged from two experiments (> 60 cells were measured for each fibroblast line). Unpaired t-test. Scale bar: 10 µm (**d**) Scatterplots of nuclear position of control and GAPO fibroblasts after two hours spreading on collagen I. The axes represent the percentage of the cell radius relative to the cell center. Quantification of the mean square displacement (MSD) of the nuclei of the indicated fibroblasts with the respective p-values are shown on the right. Unpaired t-test. Data are from three experiments, > 25 cells per fibroblast line. (**e**) Representative images of control and GAPO fibroblasts stained for F-actin (green). Cells were left untreated, treated with 25 µM latrunculin A for 30 min and fixed or fixed after 30 min and 1 h of drug washout. The graph on the right shows the fraction of cells containing stress fibers after 30 min (n = 2) or 1 h (n = 4) washout. Scale bar = 10 µm. Two-way ANOVA. (**f**) Control and GAPO fibroblasts were grown for 48 h on glass coverslips or on coverslips coated with 30 µg/ml collagen I. Cells were then fixed and stained for lamin A/C. Quantification of abnormally shaped nuclei is shown on the right. N = 3, 100 cells per experiment. Scale bar: 10 µm. Two-way ANOVA.

The nuclear lamina is physically connected to the cytoskeleton via the *L*inker of *N*ucleoskeleton and *C*ytoskeleton *C*omplex (LINC)^48^. While we observed nuclear abnormalities, no significant qualitative or quantitative alterations were seen in the three LINC complex components emerin, SUN1 and SUN2 in GAPO fibroblasts. Moreover, in contrast to HGPS fibroblasts, only bands corresponding to mature lamin A and lamin C are detected and no accumulation of non-processed lamin A/C was evident in GAPO patient fibroblasts (Fig. 3f and g). This suggests that ANTXR1 deficiency does not primarily affect the LINC complex or the nuclear lamina. However, we observed a significant increase in the number of SUN-nuclear-line-positive nuclei in GAPO fibroblasts, compared to control cells (Fig. 3h). The formation of these nuclear structures, defined as Transmembrane Actin Nuclear (TAN) lines, is induced by mechanical stress^49^. This finding suggests that mechanical coupling between the cytoskeleton and the nucleus may be altered in patient fibroblasts. It has been reported that ANTXR1 supports actin-mediated cell spreading on collagen I^16^. Consistent with this, we found that GAPO fibroblasts spread less than control cells when plated on collagen I (Fig. 4a) or on fibronectin (data not shown) and had fewer focal adhesions (Fig. 4b). Under stationary conditions, nuclei tend to localize near the cell centroid, and nuclear positioning depends, in part, on the function of the LINC complex and the actin cytoskeleton^50^. Interestingly, we observed that after spreading on collagen I for 2 h, GAPO fibroblasts had much sparser perinuclear actin filaments than control cells (Fig. 4c). Consistently, we discovered that the nuclei of GAPO patient fibroblasts tended to localize further from the cell centroid than the nuclei of control fibroblasts after 2 h of spreading on collagen I (Fig. 4d). Since nuclear dynamics during spreading depends on cell shape^51^, we also plated cells on confined circular micropatterns coated with collagen I, and observed a similar tendency of nuclei of GAPO fibroblasts to localize more distantly from the cell center when compared to control fibroblasts (Supplementary Fig. 7). Furthermore, fibroblasts of GAPO patients were impaired in reassembling stress fibers after the depolymerization of actin filaments with latrunculin A (Fig. 4e). Remarkably, when GAPO patient fibroblasts were cultured on collagen I for a 48-h period, we observed a significant improvement in nuclear morphology (Fig. 4f). This observation suggests that an altered ECM-cytoskeleton-nucleus coupling may play a significant role in determining the nuclear envelope phenotype of these cells. Furthermore, ANTXR1-deficient WI-26 cells exhibited a decreased F-actin/G-actin ratio under basal conditions, a failure to rapidly reassemble actin stress fibers following latrunculin A treatment, reduced 2D migration, and a diminished capacity to contract a collagen gel (Supplementary Fig. 8). Altogether these results suggest that ANTXR1-deficient fibroblasts have altered actin dynamics.

**Figure 5:**
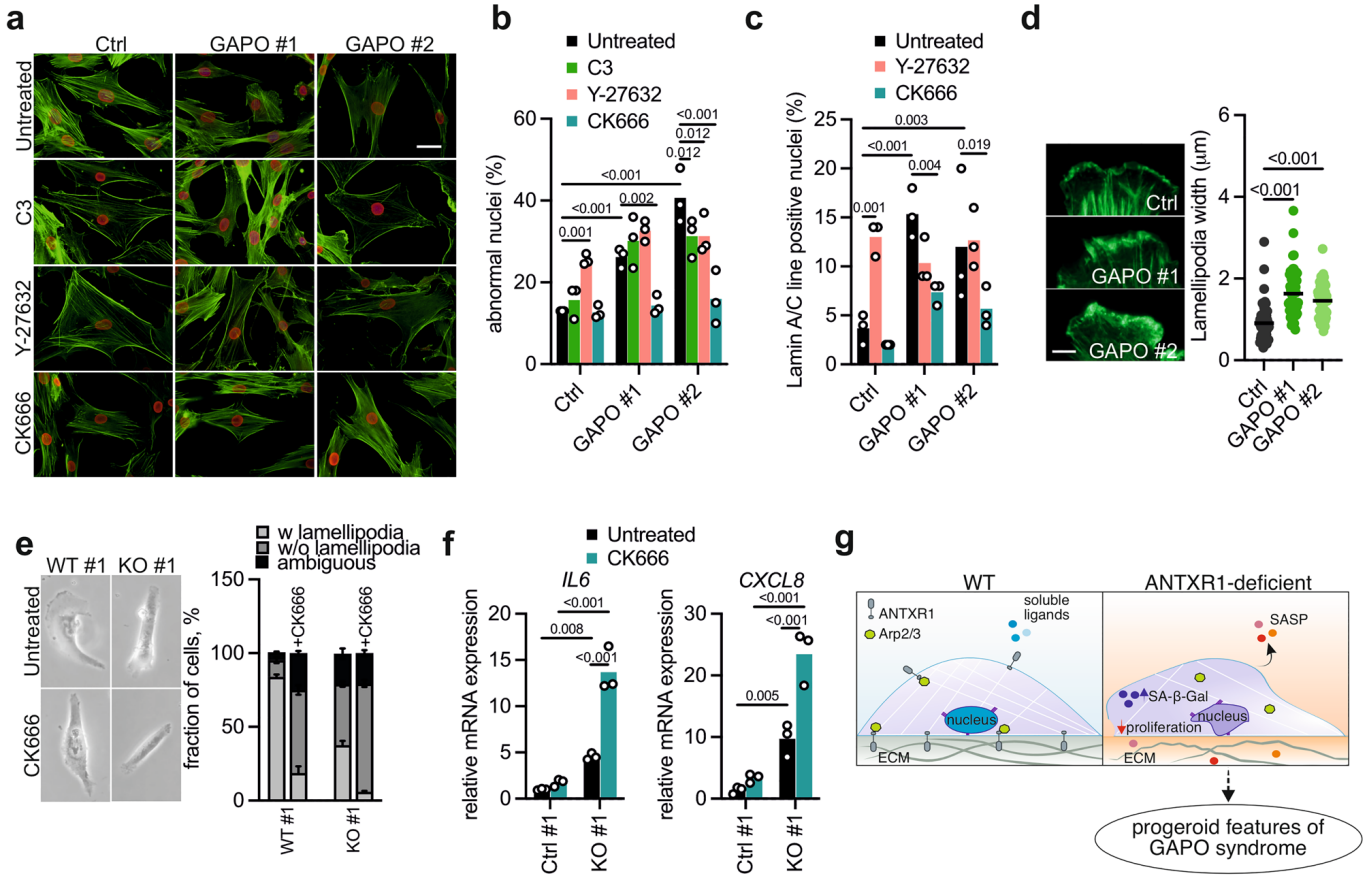
Evidence for altered Arp2/3 activity in ANTXR1-deficient fibroblasts. (**a**) Examples of fluorescence microscopy images of control and GAPO fibroblasts left untreated or treated for 48 h with the Rho inhibitor C3 (1 µg/ml), the ROCK inhibitor Y-27632 (10 µM) or the Arp2/3 inhibitor CK666 (50 µM) and stained for F-actin (green), lamin A/C (red) and with DAPI (blue). Scale bar = 20 µm. (**b**) Frequency of abnormally shaped nuclei of control and GAPO fibroblasts left untreated or treated for 48 h with the indicated actin network inhibitors. Data are from three independent experiments. For each condition 200 nuclei were evaluated in each experiment. Two way ANOVA, multiple comparisons. (**c**) Frequency of nuclear lines in untreated fibroblasts or treated for 48 h with the indicated inhibitors. For the measurements, images of lamin A/C-stained nuclei were used. Data are from three independent experiments, 100 cells per cell line per condition for each experiment. Two way ANOVA, multiple comparisons. (**d**) Exemplary images of phalloidin-stained lamellipodia (green) of control and GAPO fibroblasts. Scale bar: 5 µm. The graph on the right shows quantification of the lamellipodia width in the indicated fibroblasts after spreading for two hours on collagen I. Data are from two independent experiments. Unpaired t-test. (**e**) Representative phase contrast images of wild type and ANTXR1-deficient WI-26 cells. Cells were either left untreated or treated with the Arp2/3 inhibitor CK666 for three hours. On the right, frequency of lamellipodia in wild type and ANTXR1-deficient WI-26 fibroblasts. Cells were grown overnight on glass coverslips and left untreated or treated for three hours with 50 µM CK666 before microscopic analysis. N = 3, 100 cells per condition. (**f**) qPCR analysis of the SASP components *IL6* and *CXCL8* expression in control and ANTXR1-deficient WI-26 fibroblasts treated or left untreated for 48 h with the Arp2/3 complex inhibitor CK666 (n = 3). Two way ANOVA. (**g**) Schematic illustration summarizing the findings of this work. In physiological conditions, ANTXR1 is expressed at the cell surface, enabling cells to sense and respond to the cellular microenvironment (ECM and/or soluble ligands) by appropriately remodeling the actin cytoskeleton (partially through engagement with the arp2/3 complex) and the nuclear envelope. Conversely, in ANTXR1-deficient cells, the coupling between the microenvironment and the cytoskeleton is impaired, leading to cytoskeletal remodeling, alteration of the nuclear envelope, and induction of cellular senescence. Consequently, these cellular alterations result in the pathological reshaping of the microenvironment and the extracellular matrix through the secretion of proinflammatory factors and proteases. Taken together, these cellular events may contribute to the clinical features of GAPO syndrome.

Next, in order to investigate which of the different actin networks may specifically contribute to the nuclear defects observed in ANTXR1-deficient cells, we examined the nuclear morphology of control and GAPO fibroblasts after treating them for 48 h with inhibitors of different cytoskeletal regulators (Fig. 5a). Cytoskeletal contractility is regulated by Rho GTPases, which promote the formation of actin bundles, stress fibers, and tensile structures. Treatment with the Rho inhibitor C3 consistently resulted in the loss of stress fibers. However, it had minimal effect on the nuclear morphology of control fibroblasts and did not impact the nuclear lamina alterations observed in GAPO fibroblasts. By decreasing cytoskeletal tension with a ROCK inhibitor (Y-27632), we observed the loss of stress fibers. This inhibitor also led to an increased number of nuclei with structural defects in control fibroblasts, while it had little effect on the nuclei of GAPO fibroblasts. Finally, to investigate the role of the actin nucleator factor Arp2/3, we utilized the well-established CK666 small molecule inhibitor^52^. Treatment with CK666 induced moderate changes in cytoskeletal architecture in the different fibroblasts. Remarkably, the inhibition of Arp2/3 was able to rescue the nuclear morphology in patient cells (Fig. 5b). Interestingly, the treatment with CK666 also resulted in a reduction of the TAN lines in GAPO fibroblasts, while inhibiting ROCK increased TAN lines in control but not in GAPO fibroblasts (Fig. 5c). This finding is intriguing, particularly in light of recent studies supporting a role of Arp2/3 activity in cellular senescence^53–55^. Interestingly, treatment with CK666 caused an upregulation of *p21* in both control and GAPO fibroblasts, but not of the SASP components *IL6* and *CXCL8* (Supplementary Fig. 9). Arp2/3 nucleation activity has been found to be required for the assembly of a perinuclear actin network around the nucleus of migrating cells^56^ and for the formation of the actin cap, a cytoskeletal structure that is associated with the nuclear envelope and important for controlling the nuclear shape^53,57^. Notably, we observed that actin caps in GAPO fibroblasts are more sensitive to Arp2/3 inhibition by CK666. (Supplementary Fig. 10). Lamellipodia, sheet-like membrane protrusions composed of highly branched actin filaments, depend on the nucleation activity of the Arp2/3 complex^58^, and represent a well-established model to study its activity. Interestingly, lamellipodia were irregularly shaped in GAPO fibroblasts spread on collagen I and were on average wider compared to those of control cells. (Fig. 5d). Next, we examined the lamellipodia in ANTXR1-deficient WI-26 fibroblasts. Strikingly, quantitative analysis revealed a significantly lower proportion of cells with well-developed lamellipodia in the knockout fibroblasts. Treatment with CK666 reduced the number of lamellipodia-positive cells, confirming the crucial role of the Arp2/3 complex (Fig. 5e). We obtained similar results when analyzing a second pair of WT/KO WI-26 cells (not shown). Intriguingly, the inhibition of Arp2/3 complex activity led to an upregulation of *IL6* and *CXCL8* expression with a more pronounced effect observed in ANTXR1-deficient WI-26 fibroblasts (Fig. 5f). These findings support the hypothesis of an altered Arp2/3 activity in these cells, potentially contributing to the senescent phenotype (Fig. 5g).

## Discussion

Anthrax toxin receptors ANTXR1 and ANTXR2 are the two cell surface receptors responsible for the internalization of the anthrax toxin^15^. ANTXR1 also functions as the cellular receptor for Seneca Valley virus^2^. However, their medical significance extends beyond pathogen uptake, as pathogenic variants affecting both receptors have been linked to distinct autosomal recessive disorders. Specifically, pathogenic variants in ANTXR2 have been associated with hyaline fibromatosis syndrome, while ANTXR1 pathogenic variants are responsible for GAPO syndrome^25,59^. These disorders exhibit a multisystemic nature, impacting on various organs and yielding a diverse range of clinical manifestations, including pathologic accumulation of ECM components^38,60^. These observations underscore the critical role of ANTXRs in maintaining connective tissue homeostasis. Previous investigations utilizing mouse and cellular models have confirmed the involvement of ANTXRs as determinants of tissue architecture. These receptors appear to play a pivotal role in mediating cell–matrix adhesion, as well as coordinating the synthesis and breakdown of the extracellular matrix (ECM)^5,16,18,23,24,61–63^. However, a comprehensive characterization of their physiological functions and the mechanistic understanding of how ANTXR defects contribute to these hereditary disorders remains incomplete. In this study, by examining the consequences of ANTXR1 deficiency in human fibroblasts, we shed light on the pathogenesis of GAPO syndrome. Intriguingly, we uncover an unexpected convergence between GAPO syndrome and conditions related to premature aging.

Through an unbiased secretome analysis, we identified a notable shift toward a pro-inflammatory secretory phenotype in ANTXR1-deficient WI-26 fibroblasts. This finding prompted us to explore senescence-related markers, as the secretion of inflammatory mediators is a common characteristic of senescent fibroblasts^33^.

The discovery of ANTXR1 deficiency being associated with fibroblast senescence carries significant clinical implications. Cellular senescence is a hallmark of aging, with the accumulation of senescent cells contributing to age-related tissue degeneration, while their elimination being advantageous^43,44^. Notably, the diverse clinical features observed in GAPO patients largely mirror those seen in individuals affected by disorders characterized by accelerated aging, as also recently reported by other researchers reviewing GAPO patient phenotypes^31^. The primary features of GAPO syndrome, including growth retardation, alopecia, dental anomalies, and optic atrophy, overlap with manifestations seen in patients with progeroid disorders. Additional findings such as facial dysmorphisms, joint and bone abnormalities, skin changes, and atherosclerosis are shared among these clinical entities^31^. The mutations underlying progeroid disorders generally affect genome integrity^64^. How deficiency in ANTXR1, a plasma membrane receptor, can cause DNA damage resulting in accelerated tissue aging remains an intriguing question. ANTXR1 is characterized by the presence of an extracellular VWA domain, a transmembrane domain, and a cytosolic tail with actin-binding capabilities. Because of its structure, ANTXR1 appears to be a monomeric integrin-like receptor^15^. Integrins are considered the main cellular receptors functioning as mechanical sensors of the microenvironment by transmitting forces to the actin cytoskeleton^65^. Actin filaments are, in turn, connected to the nuclear lamina via LINC complexes^48^. Integrin-dependent force transmission from the ECM to the cytoskeleton, via the LINC complex, and the nuclear lamina influence chromatin accessibility and transcription to modulate cell states^66,67^. Growing evidence indicates that aging is associated with modifications in tissue mechanical properties and mechanosensing^53^. Moreover, genetic evidence stemming from HGPS patients harboring mutations in lamin A, one of the main structural components of the nuclear lamina, undeniably establishes a connection between the integrity of the nuclear envelope and the aging process^45^. Therefore, changes in cell-ECM interactions can drive cellular senescence and aging via LINC and nuclear envelope-dependent regulation of transcriptional activity. Like integrins, ANTXR1 has been proposed to function as an ECM receptor involved in cell adhesion and in the transmission of mechanical cues to the actin cytoskeleton^21^. Accordingly, we observed that cell spreading and actin dynamics are defective in ANTXR1-deficient fibroblasts, along with changes in nuclear architecture. Therefore, the pleiotropic clinical manifestations of GAPO syndrome may result, at least in part, from a failure to transmit forces from the ECM to the nuclear lamina. This idea seems to be corroborated by the finding that the nuclear envelope morphology of GAPO patient fibroblasts improves when cells are plated on collagen I instead of glass. Previous studies have shown that deficiency of ANTXRs leads to an abnormal ECM structure^15^. Therefore, not only could ANTXR1 function as a sensor for extracellular cues, but it could also play an active role in the remodeling of the ECM itself, as integrins also do^68^. ECM remodeling has been described as both a driver and a consequence of cellular senescence^36^. Moreover, recent evidence suggests that fibroblast senescence, specifically, plays a crucial role in tissue aging^53^. Therefore, ANTXR1 may have an essential and non-redundant role in regulating a feedback system between the ECM and stromal cells, which governs tissue architecture and aging. As the specific ligands of ANTXR1 are not fully characterized^15^, it is also possible that reduced levels of individual ECM components, rather than a broader change in the mechanical properties of the tissues, may trigger an ANTXR1-mediated switch toward a senescent phenotype in aging tissues. This raises the intriguing possibility of affecting the aging process by modulating the composition of the ECM. Similarly, physiological or pathological fluctuations in ANTXR1 levels may be crucial contributors to systemic or organ-specific age-related dysfunction. As for the extracellular VWA domain, binding partners for the cytoplasmic tail are also largely unknown, and whether ANTXR1 binds actin directly or through adaptor proteins is not resolved^15^. Previous studies have identified Rho GTPases as intracellular mediators of ANTXR1 and ANTXR2 effects on the cytoskeleton^69,70^. Interestingly, a highly conserved portion of ANTXR1 is a juxtamembrane region with homology to the Arp2/3 regulatory Wiskott-Aldrich syndrome protein (WASP)^15^. Importantly, the Arp2/3 complex is emerging as a key player in cellular senescence^53,54^. Indeed, observed functions of the Arp2/3 complex in the regulation of perinuclear actin dynamics^56^, DNA repair^71^ and cell division^54^ indicate a multifaceted role in processes interconnected with cellular senescence.

In summary, we have identified a role for ANTXR1 in preventing senescence in human fibroblasts, suggesting that GAPO syndrome may be reclassified as a progeroid disorder.

Our research on GAPO syndrome contributes to the body of evidence from recent studies^53,72–74^ linking the ECM and cell–matrix adhesion to premature aging phenotypes.

## Materials and methods

### Cell culture, transfections, and treatments

All cells were cultured in DMEM/F12 supplemented with 10% FBS and 1% penicillin/streptomycin and controlled for mycoplasma contamination. WI-26 cells were obtained from the American Type Culture Collection (ATCC; CCL-95.1). The two GAPO patient primary dermal fibroblast lines have been described before^25^. Control fibroblast cultures were established by outgrowth from skin biopsies of 8-, 20-, 53- and 96-year-old healthy donors as described^75^. Primary fibroblasts were used from passage 5 to passage 20. ANTXR1-deficient WI-26 were generated using the CRISPR/Cas9 system and the PX459 vector^76^. sgRNA sequences were: sense: 5´-CACCGCATTAAGGTTGTTCCTCGGG-3´; antisense: 5´-AAACCCCGAGGAACAACCTTAATGC-3´. WI-26 cells were transfected, and selected with puromycin (1 μg/ml for 2 days). Single cell clones were picked using cloning cylinders (Thermo Fisher Scientific), further expanded and analysed by immunoblotting, qPCR and DNA sequencing. For ECM production cells were kept in serum free medium supplemented with ascorbate for four days. Medium was changed every other day. siRNAs were reverse transfected using Lipofectamine RNAi MAX (Invitrogen). siRNA sequences targeting *ANTXR1* were: #1: 5´-ATCCGTCAAGGCCTAGAAGAA-3´ and #2: 5´ CTCGGTCACACTCAATGAGAA 3´^41^. AllStars Negative Control siRNA (Qiagen) was used as a transfection control. For the pharmacological regulation of cytoskeletal contractility and dynamics, primary fibroblasts were treated with C3 Transferase protein (Cytoskeleton Inc.), Y-27632 (Adipogen Life Sciences), CK666 (Tocris) at the indicated concentrations. The same volume of DMSO was used as a control treatment.

### Mass spectrometry analysis

To generate conditioned media for secretome analysis, 2.5 × 10^5^ cells were seeded in 6-well plates, grown for 48 h and then shifted on serum free medium for four more days. Ascorbate was added every other day. The medium was collected, acetone precipitated, in-solution digested and loaded onto SDB-RP StageTips according to facility protocols.

All samples were analyzed by the CECAD proteomics facility on a Q Exactive Plus Orbitrap mass spectrometer coupled to an EASY nLC (both Thermo Fisher Scientific). Peptides were loaded with solvent A (0.1% formic acid in water) onto an in-house packed analytical column (50 cm, 75 µm inner diameter, filled with 2.7 µm Poroshell EC120 C18, Agilent). They were chromatographically separated at a constant flow rate of 250 nL/min using the following gradient: 4–6% solvent B (0.1% formic acid in 80% acetonitrile) within 5.0 min, 6–23% solvent B within 120.0 min, 20–54% solvent B within 7.0 min, 54–85% solvent B within 6.0 min, followed by washing and column equilibration. The mass spectrometer was operated in data-dependent acquisition mode. The MS1 survey scan was acquired from 300 to 1750 m/z at a resolution of 70,000. The ten most abundant peptides were isolated within a 2.1 Th window and subjected to HCD fragmentation at a normalized collision energy of 27%. The AGC target was set to 5e5 charges, allowing a maximum injection time of 60 ms. Product ions were detected in the Orbitrap at a resolution of 17,500. Precursors were dynamically excluded for 25.0 s. All mass spectrometric raw data were processed with Maxquant (version 1.5.3.8, Ref.^77^) using default parameters against the Uniprot canonical Human database (UP5640, downloaded 20.01.2022) with the match-between-runs option enabled between replicates. Follow-up analysis was done in Perseus 1.6.15^78^. Hits from the decoy database, the contaminant list and those only identified by modified peptides were removed. Afterwards, results were filtered for data completeness in replicates groups and LFQ values imputed using sigma downshift with standard settings. Finally, FDR-controlled T-tests between sample groups were performed with s0 = 0.2.

### qPCR

RNA extraction was carried out with TRIzol™ following the manufacturer´s protocol. Total RNA (1–2 μg per sample) was reverse transcribed using the OmniScript RT Kit (Qiagen). Technical duplicates for every sample were amplified using the Takyon ROX SYBR 2X MasterMix dTTP blue (Eurogentec) either in a StepOnePlusTM real-time PCR detection system (Applied Biosystems) or in a LightCycler 480 (Roche). Data analysis was performed using the 2^−ΔΔCt^ method and quantified relative to *GAPDH*. Primer sequences are provided in Supplementary Table 2.

### Immunofluorescence and microscopy

Cells were seeded onto 12 mm glass cover slips in 24-well plates for 24–48 h and fixed with 4% PFA/PBS for 15 min. For ECM network formation, 5 × 10^4^ cells were seeded on glass cover slips and grown for five days and ascorbate added every second day and then fixed with an ice-cold methanol/acetone solution at − 20 °C for 10 min. PFA fixed cells were permeabilized with PBS/0.5% NP-40 for 10 min. Cells were blocked with 1% FBS in PBS for 30 min prior to incubation with primary antibodies for 1 h at RT. Primary antibodies are listed in Supplementary Table 3. After washing, the samples were incubated with appropriate, highly cross-adsorbed, secondary antibodies conjugated to AlexaFluor 488, 555 or 647 (Thermo Fisher Scientific) or/and with AlexaFluor-488 or 647 conjugated phalloidin (Cell Signaling, Thermo Fisher Scientific) and DAPI (0.1 µg/ml; Sigma-Aldrich) for 1 h at RT. Images were taken with a Leica TCS SP5 confocal microscope, a Zeiss Axiophot fluorescent microscope or with a Nikon Eclipse 80i upright fluorescence microscope. Phase contrast images of live cells were taken with a Zeiss Primovert mounted with an Axiocam 208 color camera.

### Cell lysis and western blotting

2.5 × 10^5^ cells were seeded in 6-well plates and grown for a minimum of 48 h. For the detection of ECM proteins, cells were shifted to serum free medium for 4 days. Ascorbate was added every other day. The cells were lysed in RIPA buffer (20 mM Tris/HCl pH 7.4, 150 mM NaCl, 1% NP-40, 0.05% Triton X-100, 0.5% SDS). The lysates were sonicated and centrifuged. For G- and F-actin detection, 2.5 × 10^5^ cells were seeded in 6-well plates and grown for 48 h. The cells were then processed using the G-actin / F-actin In Vivo Assay Kit (Cytoskeleton, Inc.) according to the manufacturer´s instructions. Quantification of western blotting was performed by calculating the intensity of protein bands by densitometry analysis using the Fiji software.

### Proliferation assay

1 × 10^4^ cells were seeded in 12-well plates and grown at 37 °C. Every 24 h, triplicate samples were counted twice with a DeNovix CellDrop Brightfield Cell Counter.

### EdU proliferation assay

1 × 10^4^ cells were plated on glass coverslips and grown for 24 h. WI-26 cells and primary dermal fibroblasts were treated with 10 µM EdU for 1–3 h respectively, prior to fixation with 4% PFA/PBS. The coverslips were then processed using the Cell Proliferation Kit III (PromoKine) according to the manufacturer’s protocol.

### SA-β-Gal assay

SA-β-Gal staining was performed using the Senescence β-Galactosidase Staining Kit (#9860, Cell Signaling) according to manufacturer’s protocols. Briefly, 1 × 10^4^ cells were plated in 24-well plates and grown for 2–4 days prior to fixation with 4% PFA/PBS. 250 µl freshly prepared β-galactosidase solution (pH 6.0) was added per well. The plates were incubated overnight at 37 °C. Cells were shifted to a 70% glycerol solution and counted.

### Casein gel zymography

1 × 10^5^ cells were seeded in 12-well plates and grown until confluency when the medium replaced with serum free medium and the cultures incubated with 1 µg/ml plasminogen, 1 µg/ml aprotinin and with or without 0.5 mM amiloride for 1 h at 37 °C. The cells were then washed two times with serum free medium, lysed in 140 µl 1% NP-40/PBS, the lysates kept on ice for 10 min, briefly centrifuged, and subjected to electrophoresis on 10% SDS–polyacrylamide gels supplemented with 1.3 mg/ml casein. After electrophoresis, gels were washed three times with 2.5% Triton X-100/H_2_O for 15 min each to remove SDS, and submerged in development buffer (50 mM Tris/HCl pH 7.4, 10 mM CaCl_2_, 0.02% NaN_3_) at 37 °C for 18 h.

Developed gels were stained overnight with a 0.02% Coomassie solution containing 5% aluminium sulfate-(14–18)-hydrate, 10% ethanol and 2% orthophosphoric acid. A solution of 10% ethanol and 2% orthophosphoric acid was used for destaining.

### Cell spreading

Glass coverslips were coated with 30 µg/ml rat tail collagen I (Corning) or 10 µg/ml human fibronectin (Roche) in PBS overnight at 4 °C. 1 × 10^4^ Cells in serum free medium were plated and left to settle at 37 °C for the indicated time points. Cells were fixed in 4% PFA/PBS. Afterwards, the spread cells were visualized by AlexaFluor-488 conjugated phalloidin (Cell Signaling), DAPI and vinculin staining. Micropatterned glass slides were from Cytoo.

### Wound healing assay

4 × 10^4^ wilde type and ANTXR1 deficient WI-26 cells were plated into each well of 2-well culture-inserts (Ibidi) in 70 µl of medium. The next day the inserts were carefully removed. After washing the cells once, images were acquired immediately and after 24 h using a Zeiss Primovert mounted with an Axiocam 208 color camera. Quantification of the wound closure area was performed using ImageJ.

### Collagen lattice contraction

The collagen mix was prepared as follows: 1 × DMEM, 10% FBS, 1% PenStrep, 2 mM Glutamine, 50 µgml Na-ascorbate, 0.3 mg/ml rat tail collagen I (Corning), and 3% 0.1 N NaOH to neutralize the collagen solution acidity. 3 × 10^5^ cells were suspended in the collagen mix and pipetted into a bacteriological dish with untreated plastic surface. The dishes were then placed in an incubator at 37 °C and left undisturbed for at least 3 h to allow the formation of a collagen disc. After the indicated time points, images of the dish bottoms were taken via a transmitted light scanner.

### Quantifications and statistical analysis

Data are shown as indicated in the figure legends. The nuclear envelope morphology was evaluated independently by different investigators on cells stained for the different nuclear envelope components using widefield fluorescent microscopes. A minimum of 100 cells were counted for each sample. For all the image-based quantifications ImageJ/Fiji was used. Focal adhesions were quantified as in Ref.^79^. Nuclear positioning was measured by processing confocal images of fibroblasts that were allowed to spread for 2 h on collagen I and labeled with fluorescent phalloidin and DAPI. Cell and nucleus centroids were identified and the median square displacement (MSD) was calculated as in Ref.^50^. Actin intensity in the perinuclear region was measured by tracing the nuclear area as region of interest to calculate the mean fluorescent intensity of the actin filaments from widefield microscopy images, similarly as in Ref.^80^.

Statistical significance was tested using unpaired two-tailed Student’s t-test or two way ANOVA. The tests were performed under the assumption that values follow a normal distribution and have similar variance. Only statistically significant p-values (< 0.05) are shown on graphs. The tests were performed using Prism v.9.

### Supplementary Information

Supplementary Figures.

Supplementary Information.

Supplementary Table 1.

Supplementary Table 2.

Supplementary Table 3.

## Data Availability

All data supporting the findings of this study are available within the paper and its Supplementary Information. Secretome data of wild type and ANTXR1-deficient WI-26 cells is reported in Supplementary Table 1.
