# Supplementary material for: ANTXR1 deficiency promotes fibroblast senescence: implications for GAPO syndrome as a progeroid disorder
Source: Sci Rep. 2024 Apr 23;14:9321. doi: 10.1038/s41598-024-59901-y (PMC11039612; doi:10.1038/s41598-024-59901-y)
Supplement: Supplementary file 1 — Supplementary Figures. [file 41598_2024_59901_MOESM1_ESM.pdf]

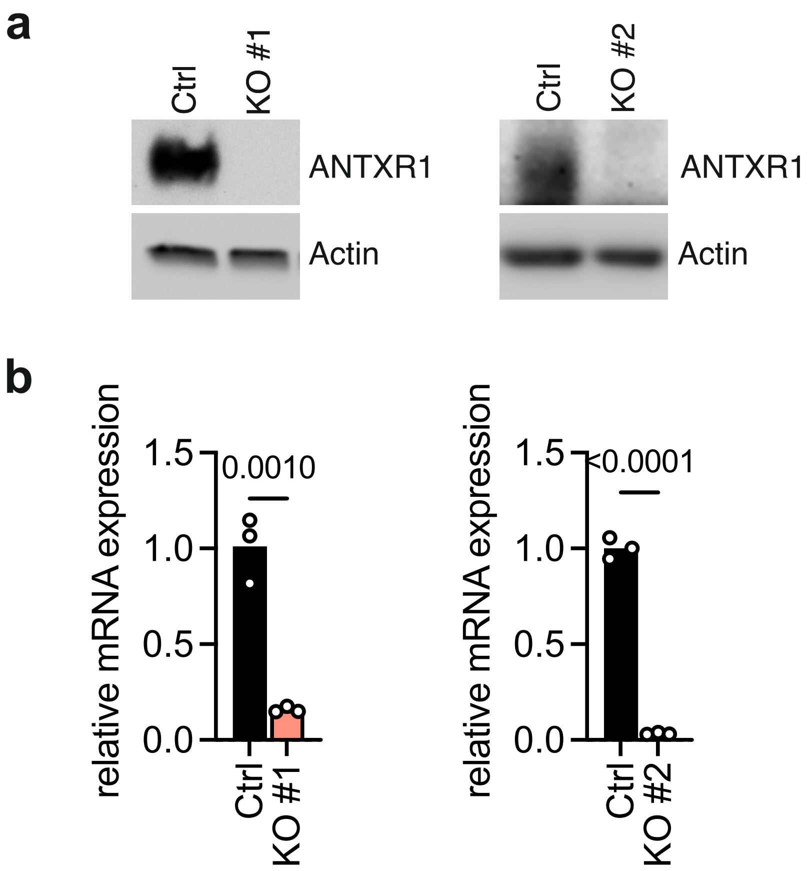

Supplementary Figure 1. Validation of ANTXR1-deficient WI-26 clones. (**a**) Cell lysates of control and ANTXR1-deficient WI-26 cells were analyzed by SDS-PAGE and detected with a monoclonal antibody against ANTXR1. Actin was used as a loading control. (**b**) *ANTXR1* mRNA expression in control and ANTXR1-deficient WI-26 fibroblasts analyzed by qPCR. Unpaired t-test.

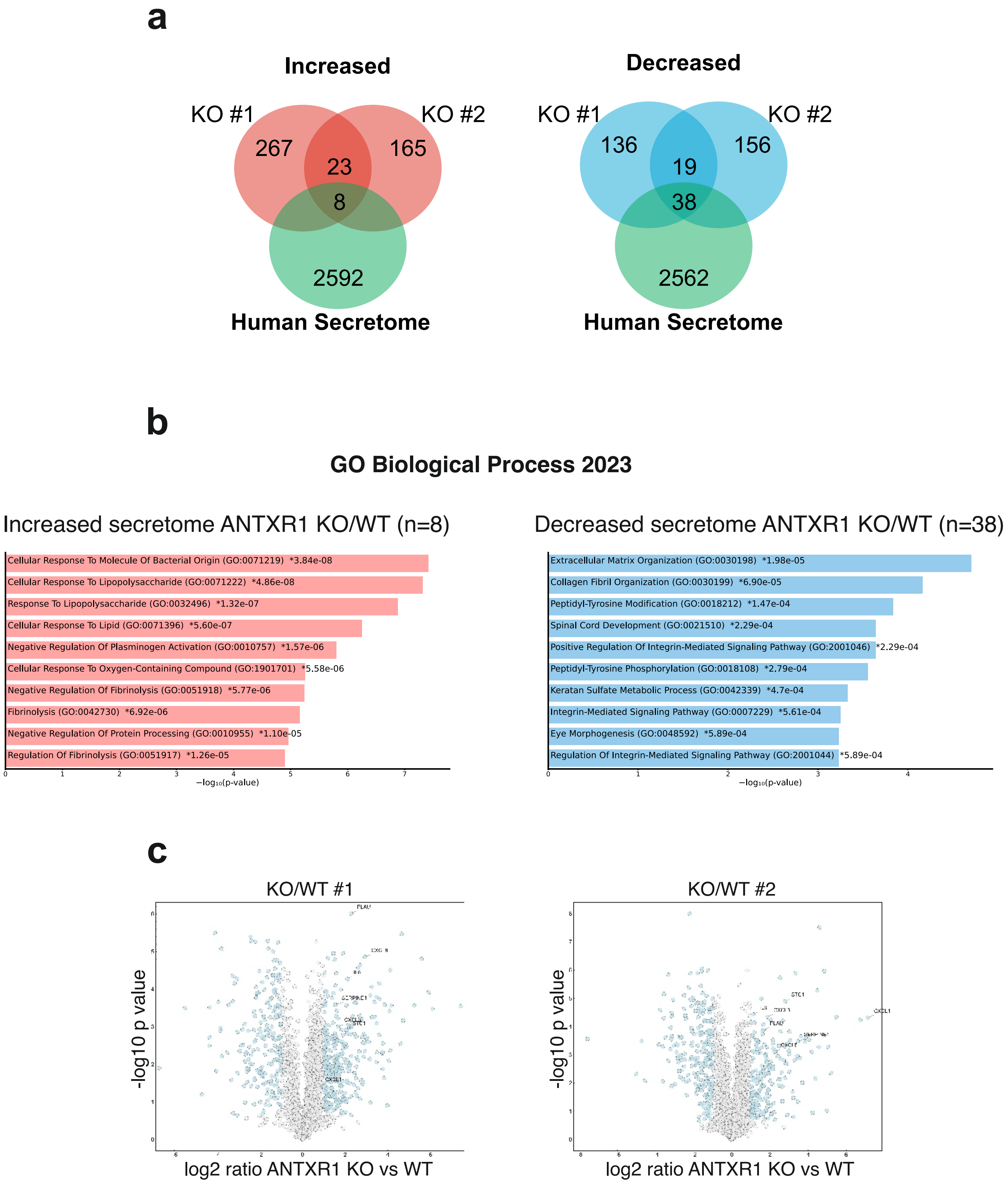

Supplementary Figure 2. Secretome analysys of ANTXR1-deficient WI-26 fibroblasts. (**a**) Venn diagrams illustrating the correlation of proteins significantly increased and decreased (log2 fold change ≥/≤ 1, p-value < 0.05) in two independent ANTXR1-deficient and control clones and their distribution within the human secretome dataset. (**b**) Gene ontology term enrichment of the 8 secreted proteins significantly increased or the 38 proteins decreased in the secretome of ANTXR1-deficient WI-26 cells. The graphs display the ten most significantly over-represented biological process terms ranked according to p-values (x-axis). Analysis was performed using Enrichr. (**c**) Volcano plots comparing protein fold changes between control and ANTXR1-deficient cells. Significantly regulated proteins are labeled in blue. Text labels indicate the SASP components that are upregulated in both secretomes.

Supplementary Figure 2

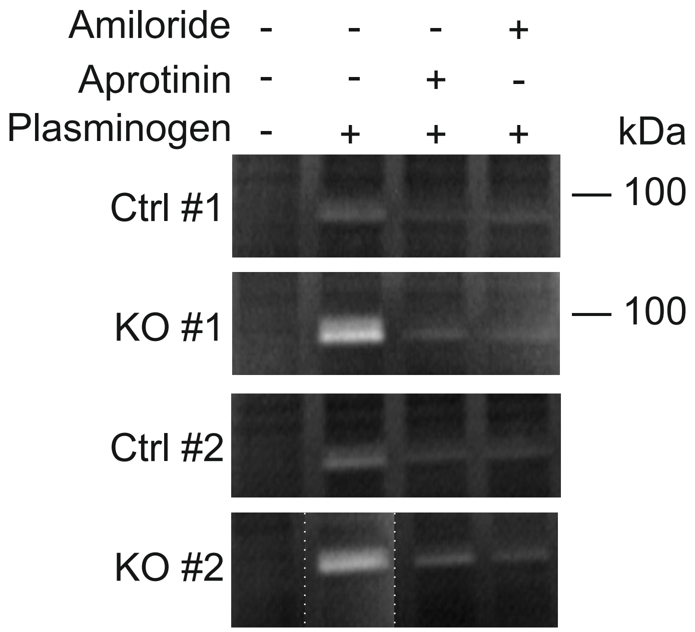

Supplementary Figure 3. Casein-plasminogen zymography of wild type and ANTXR1-deficient WI-26 cells. Cell lysates of control and ANTXR1-deficient WI-26 cells were analyzed by SDS-PAGE casein-zymography. The cells were treated with the indicated reagents for 1 h prior to lysis. Note the increased caseinolytic activity of the cell lysates of ANTXR1-deficient cells when compared to wild type cells. Inhibitor treatments confirmed that the plasminogenic activity was specifically due to increased levels of active PLAU in the cell lysate of ANTXR1-deficient cells. The KO #2 image has been cropped from the same gel to keep the loading order uniform to the other samples. Original uncropped immunoblots are presented in the Supplementary Information file.

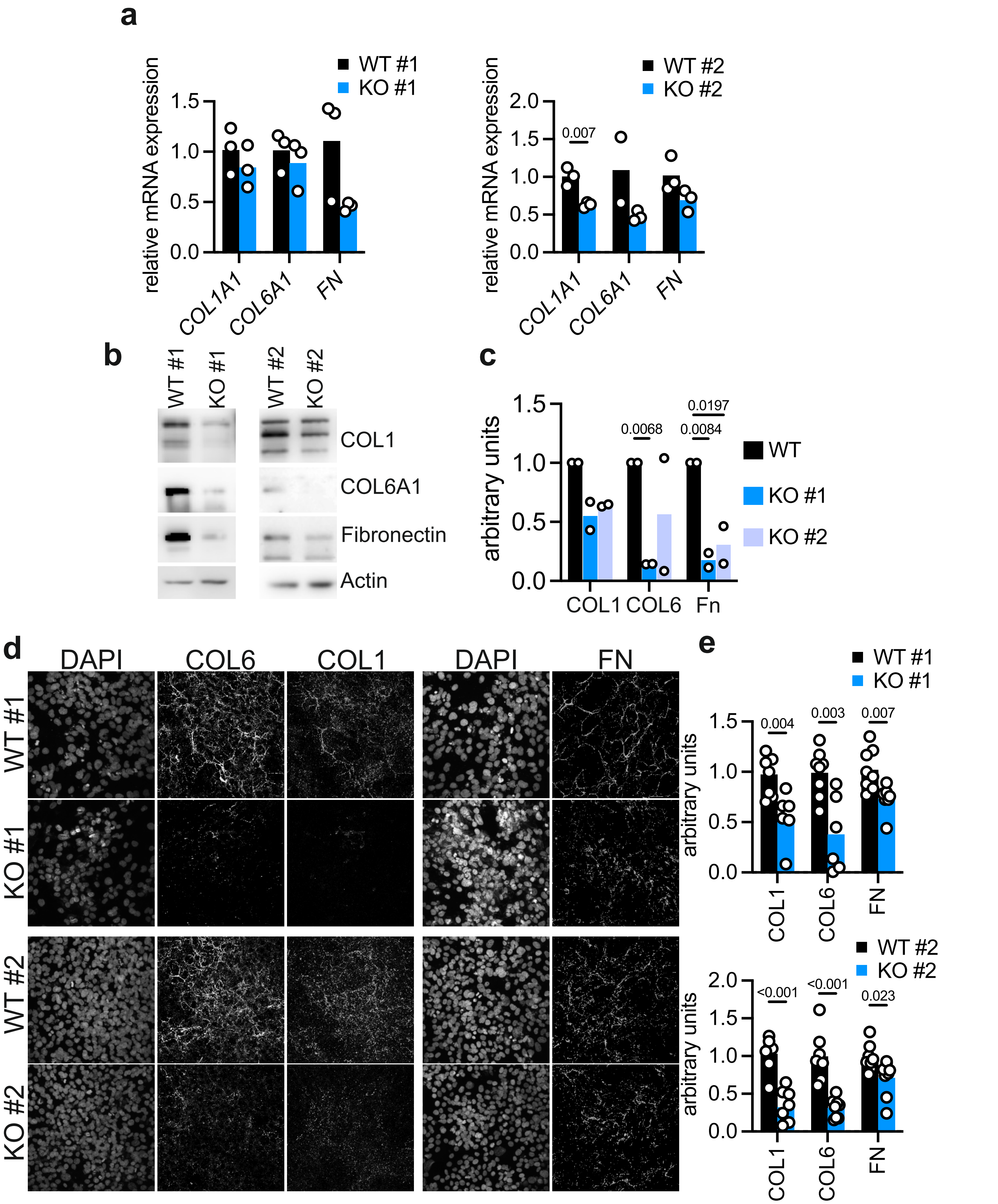

Supplementary Figure 4. Defective ECM production by ANTXR1-deficient WI-26 fibroblasts (**a**) qPCR analysis of the expression levels of the indicated genes in control and ANTXR1-deficient WI-26. Unpaired t-test (**b**) Immunoblots of ECM proteins secreted in the serum free medium of the indicated WI-26 cell lines. Original uncropped immunoblots are presented in the Supplementary Information file. (**c**) Quantification of the secreted protein detected by immunoblot as in b. Two-way ANOVA. Data were normalized to actin in the cell lysate. N=2 (**d**) Cells were grown for four days in ascorbate-supplemented serum-free medium and then immunostained with the indicated antibody to visualize extracellular matrix network formation in control and ANTXR1-deficient WI-26 fibroblasts (**e**). Quantification of the stainings as in d. Unpaired t-test.

Supplementary Figure 4

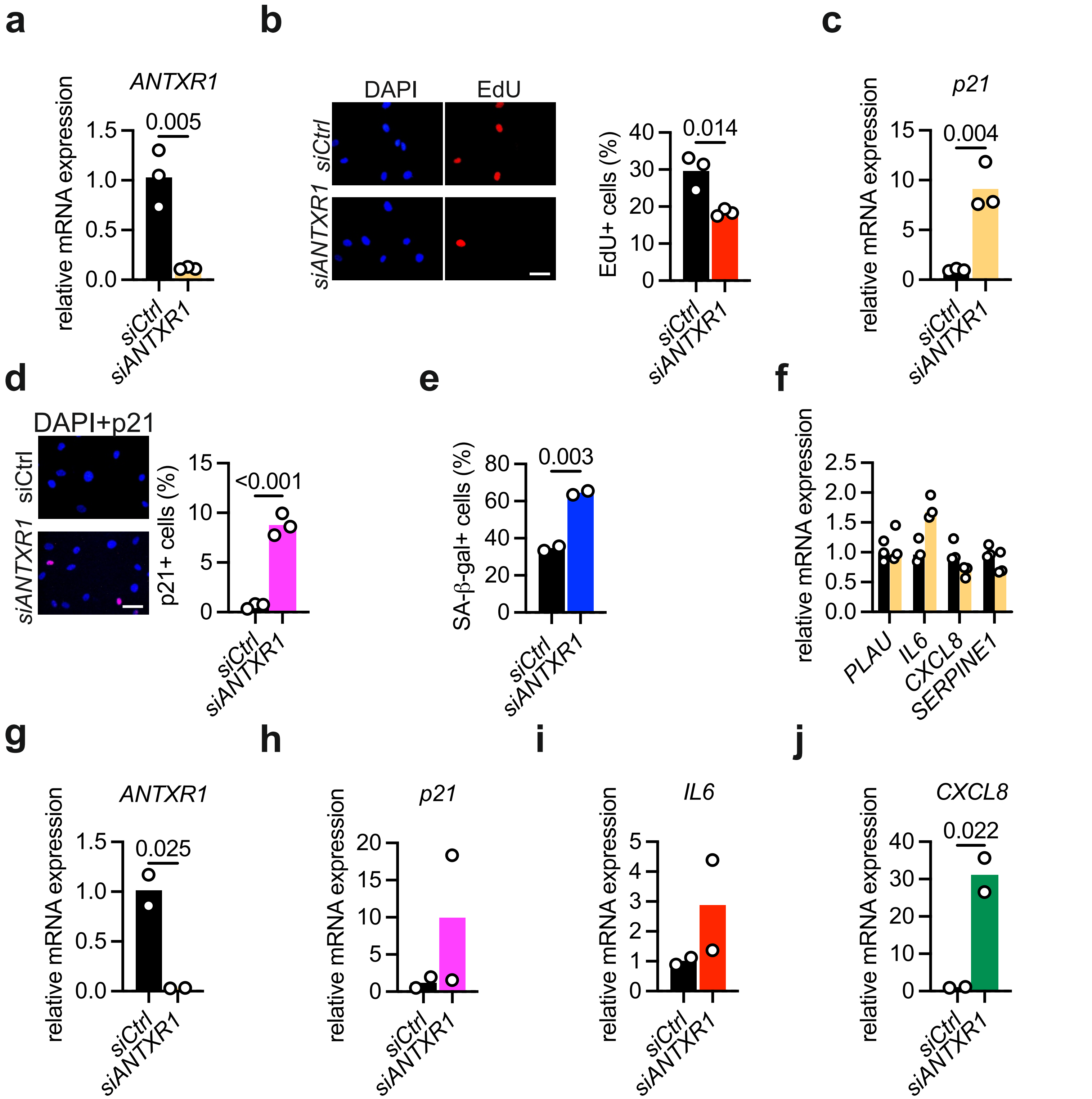

Supplementary Figure 5. ***ANTXR1* knockdown induces a senescence phenotype in primary fibroblasts** Analysis of primary human dermal fibroblasts from a 53-year-old-donor (**a-f**) and a 96-year-old donor (**g-j**) four days after reverse transfection with control siRNA or with a pair of siRNAs targeting *ANTXR1*. (**a**) RNA was isolated and *ANTXR1* mRNA expression quantified by qPCR. Unpaired t-test. (**b**) EdU incorporation in the transfected fibroblasts (n=3). >100 cells were analyzed for each sample. Unpaired t-test. (**c**) *p21* mRNA levels in the transfected fibroblasts as assessed by qPCR. Unpaired t-test (**d**) Representative immunofluorescence images and quantification of p21 positive nuclei (magenta) in the transfected fibroblasts (n=3). Unpaired t-test. (**e**) Induction of SA-β-Gal expression in the transfected fibroblasts (n=2). Each dot on the bars represents the results of one microscopic field. Five microscopic fields were analyzed for each experiment. Unpaired t-test. (**f**) Knockdown of *ANTXR1* in the fibroblasts from the 53-year-old-donor did not cause the induction of a SASP, as revealed by qPCR analysis (n=3). (**g-j**) In contrast, *ANTXR1* knockdown (**g**) in primary human fibroblasts derived from the 96-year-old-donor indicated SASP induction by displaying elevated *p21* (**h**), *IL6* (**i**), and *CXCL8* (**j**) expression as revealed by qPCR analysis. Unpaired t-test..

Supplementary Figure 5

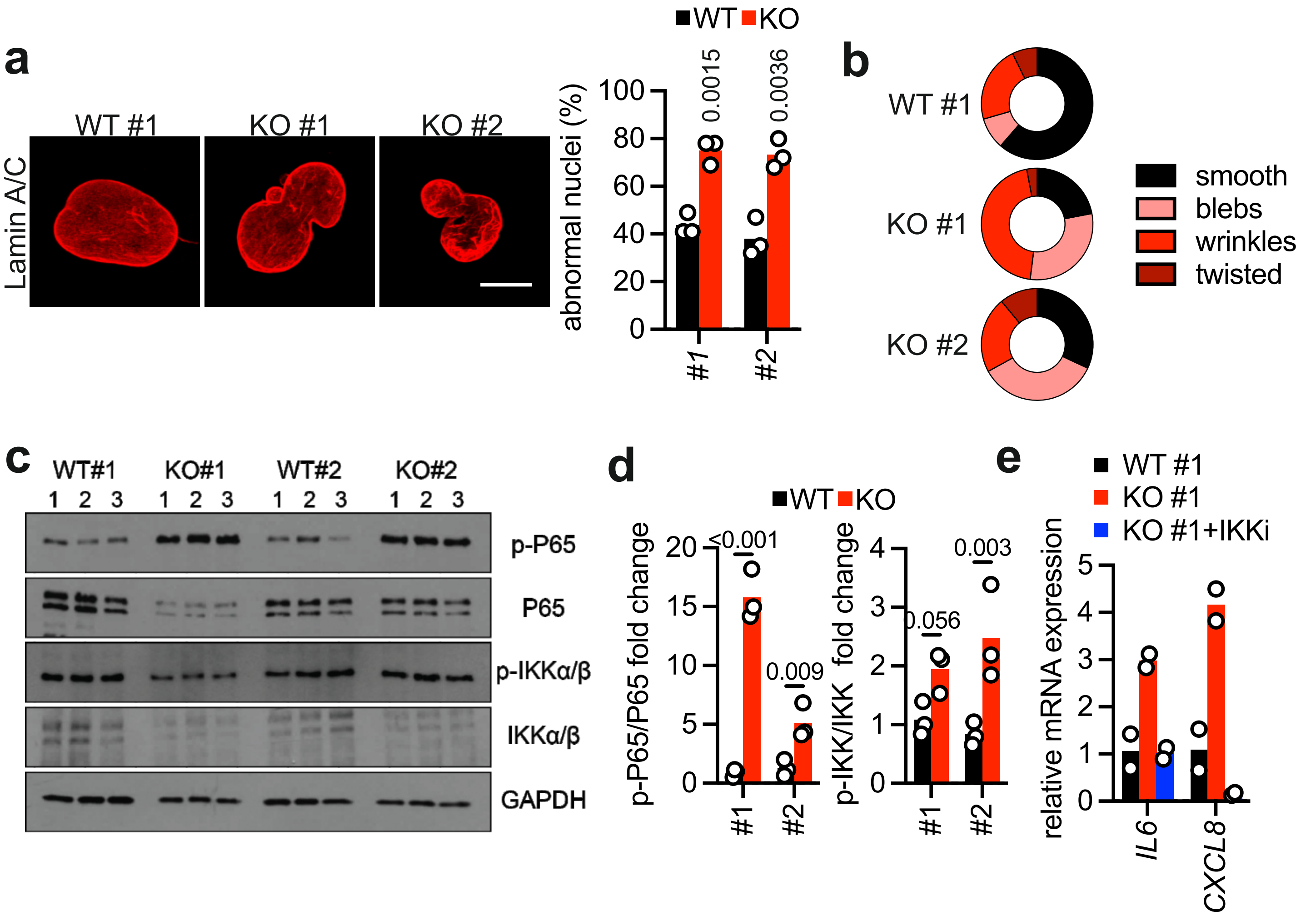

Supplementary Figure 6. Loss of ANTXR1 in WI-26 fibroblasts is associated with abnormal nuclear morphology and activation of NF-kB (**a**) Representative confocal images of control and ANTXR1-deficient WI-26 nuclei immunostained for lamin A/C. The diagram shows the frequency of abnormal nuclei in the different cell populations from three independent experiments (>100 nuclei per experiment). (**b**) The diagram shows the relative percentage of different nuclear lamina defects in control and ANTXR1-deficient WI-26 cells. (**c**) Immunoblots for p-p65 (Ser 536) and p-IKK α/β in the cell lysates of control and ANTXR1-deficient WI-26 fibroblasts. (**d**) Quantification of the p-p65 and p-IKK α/β band intensities normalized to total p65 and IKK α/β and expressed as fold change over control.Two-way ANOVA. (**e**) qPCR for selected SASP components in control and ANTXR1-deficient WI-26 fibroblasts treated with an IKK inhibitor.

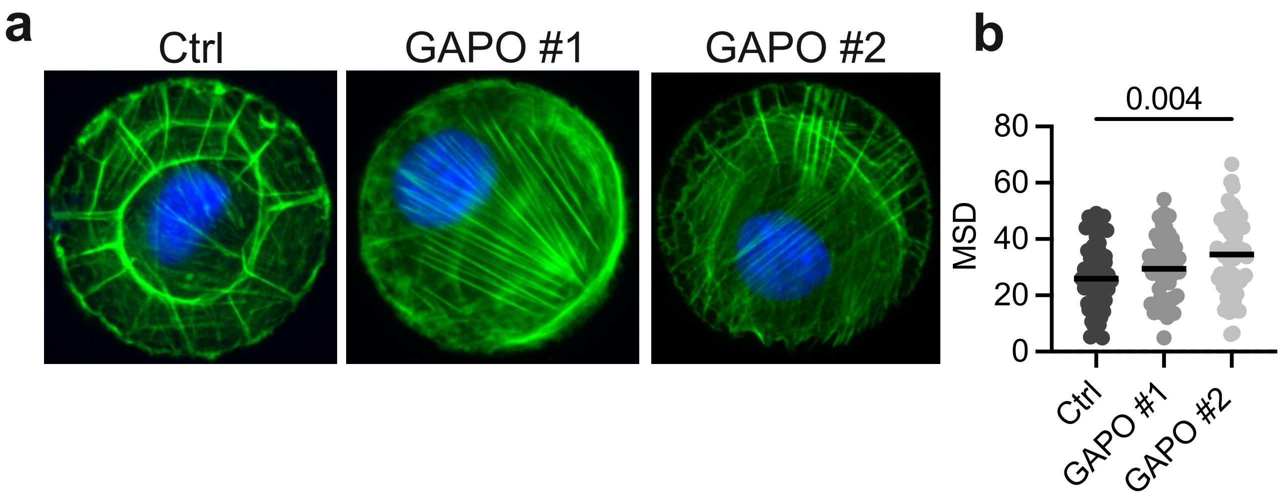

Supplementary Figure 7. Nuclear positioning in fibroblasts plated on circular micropatterns (**a**) Representative images of control and GAPO fibroblasts stained with 488-phalloidin and DAPI after 3 hours spreading on collagen I-coated circular micropatterns (700 µm2 spreading surface). (**b**) Quantification of the mean square displacement (MSD) of the nuclei of the indicated fibroblasts. Data are from 2 independent experiments, >25 cells per fibroblast line per experiment. Unpaired t-test.

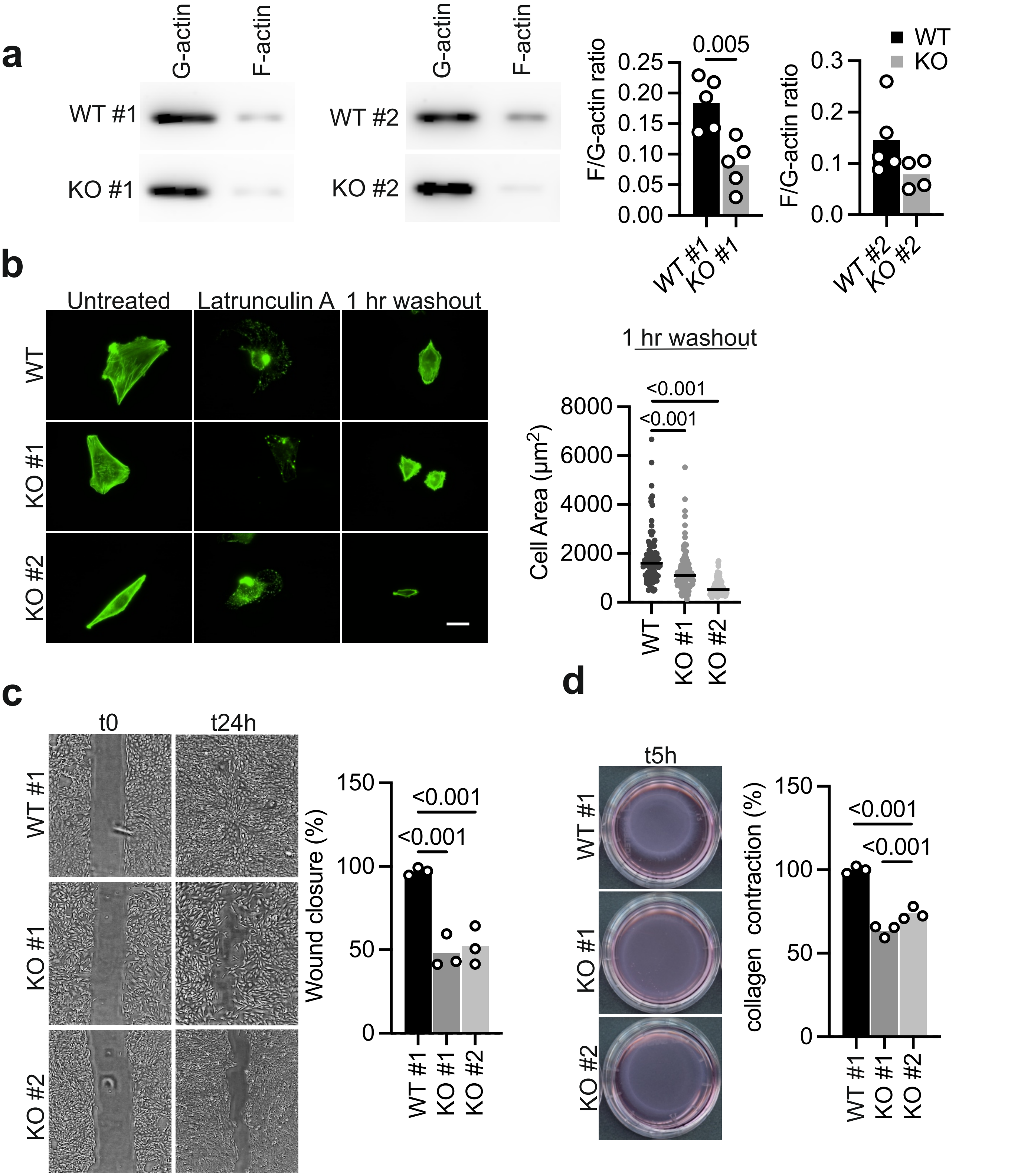

Supplementary Figure 8. Altered actin dynamics in ANTXR1-deficient WI-26 fibroblasts. (**a**) Immunoblot analysis of G and F-actin in confluent WI-26 fibroblasts. The graph on the right shows the densitometric quantification of the F/G actin ratio. n=5, unpaired t-test. Original uncropped immunoblots are presented in the Supplementary Information file. (**b**) Representative widefield microscopy of wildtype and ANTXR1-deficient WI-26 cells stained for F-actin using 488-conjugated phalloidin (green). Cells were left untreated, treated with 25 µm latrunculin A for 30 min or fixed after one hour of drug washout. On the right, quantification of cell area after one hour washout. Data are from two independent experiments, unpaired t-test. Scale bar = 10 µm (**c**) Representative images of wild type and ANTXR1-deficient WI-26 cells at time 0 and after 24 hours of incubation in serum free medium. Percent wound closure of ANTXR1-deficient WI-26 fibroblasts relative to wild type cells are shown on the right. Unpaired t-test. (**d**) Representative images of collagen I gels after 5 hours contraction by wild type and ANTXR1-deficient WI-26 fibroblasts. The graph on the right shows the quantification of three experiments. Unpaired t-test.

Supplementary Figure 8

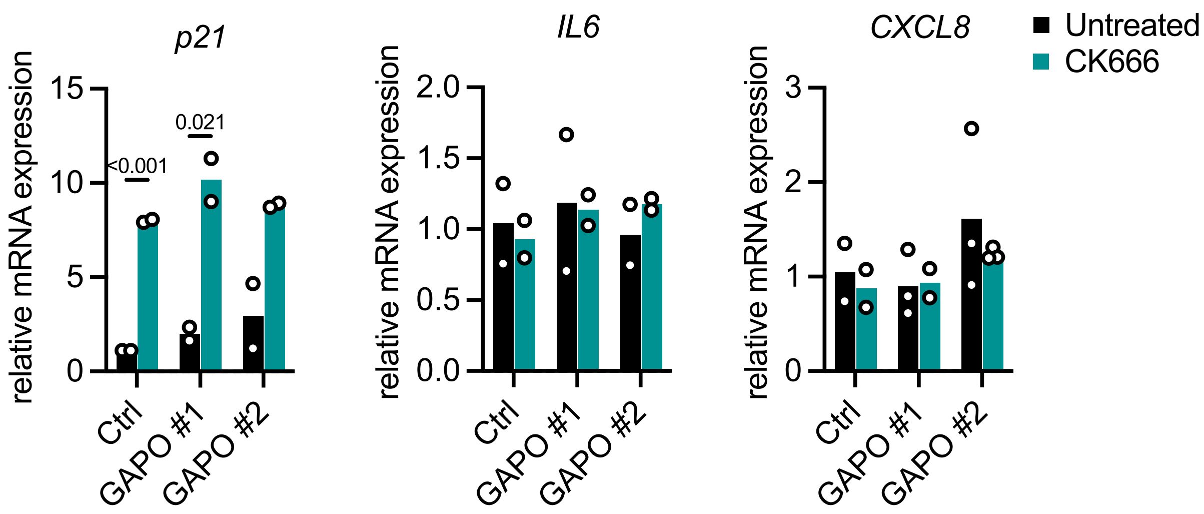

Supplementary Figure 9. Arp2/3 complex inhibition effect on p21, IL6 and CXCL8 expression in primary fibroblasts. qPCR analysis of *p21, IL6* and *CXCL8* expression in control and GAPO fibroblasts treated or not treated for 48 hours with the Arp2/3 complex inhibitor CK666 (n=2) Unpaired t-test.

Supplementary Figure 9

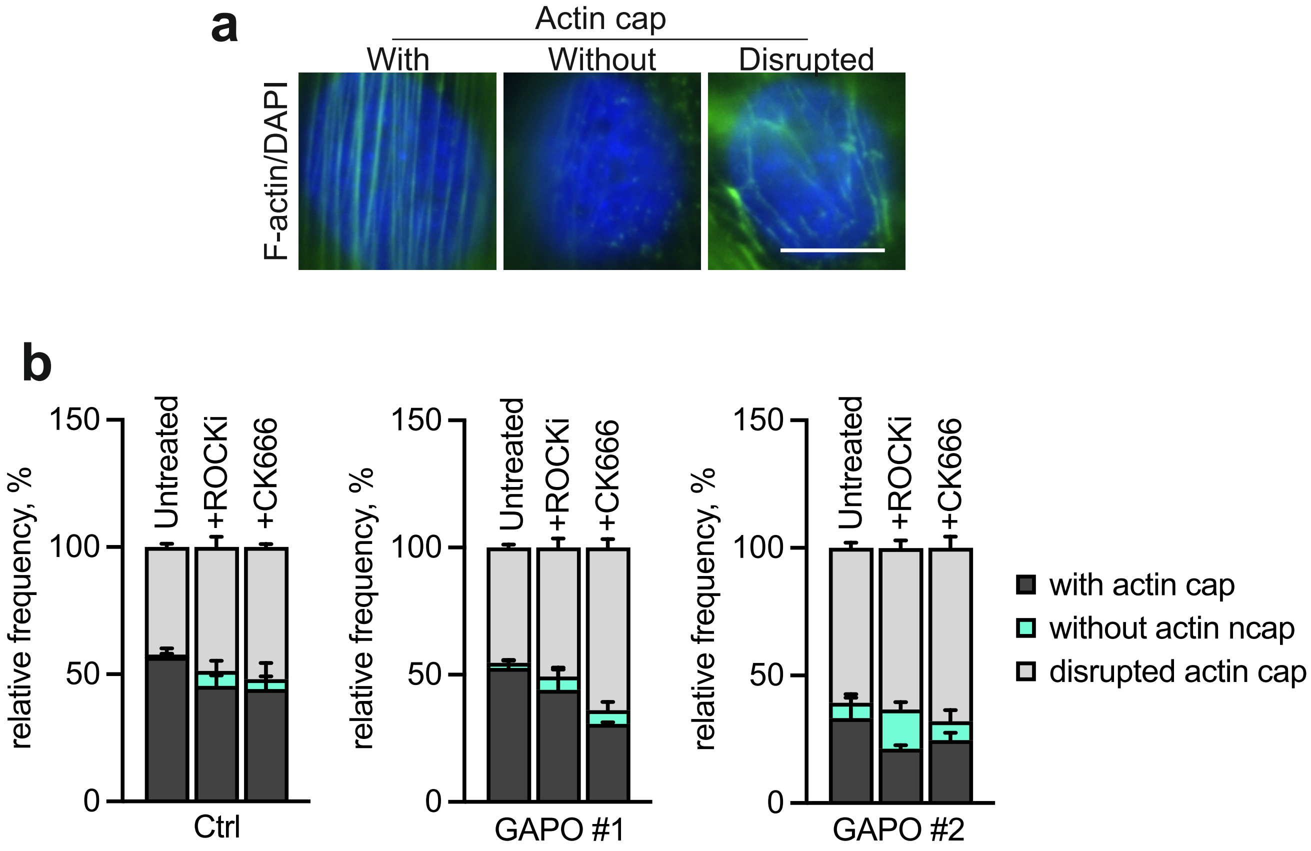

Supplementary Figure 10. Perinuclear actin cap assesment in control and GAPO patient primary fibroblasts. (**a**) Representative images of F-actin staining at the apical perinuclear actin cap of primary dermal fibroblasts. Scale bar: 10 µm (**b**) Fraction of cells showing either an organized, disorganized, or no actin cap in the indicated fibroblasts. Cells were grown on glass coverslips and either left untreated or treated for 48 hours with the ROCK inhibitor Y-27632 (10 µM) or CK666 (50 µM) to inhibit Arp2/3.
