# Supplementary material for: ANTXR1 deficiency promotes fibroblast senescence: implications for GAPO syndrome as a progeroid disorder
Source: Sci Rep. 2024 Apr 23;14:9321. doi: 10.1038/s41598-024-59901-y (PMC11039612; doi:10.1038/s41598-024-59901-y)
Supplement: Supplementary file 2 — Supplementary Information. [file 41598_2024_59901_MOESM2_ESM.pdf]

Supplementary Figure 1

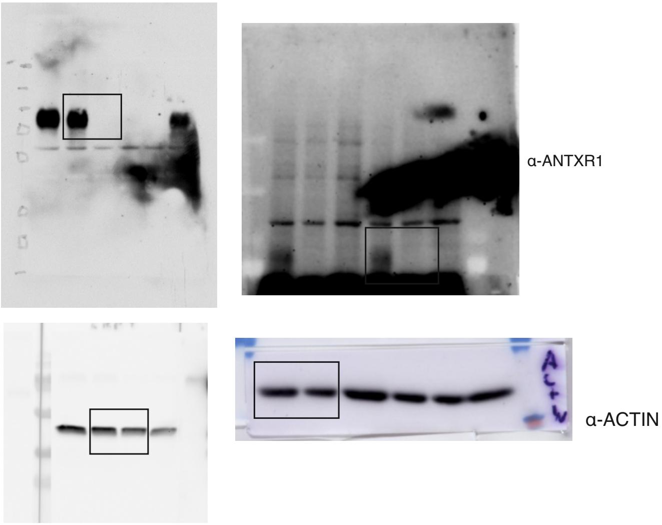

Fig. 1e

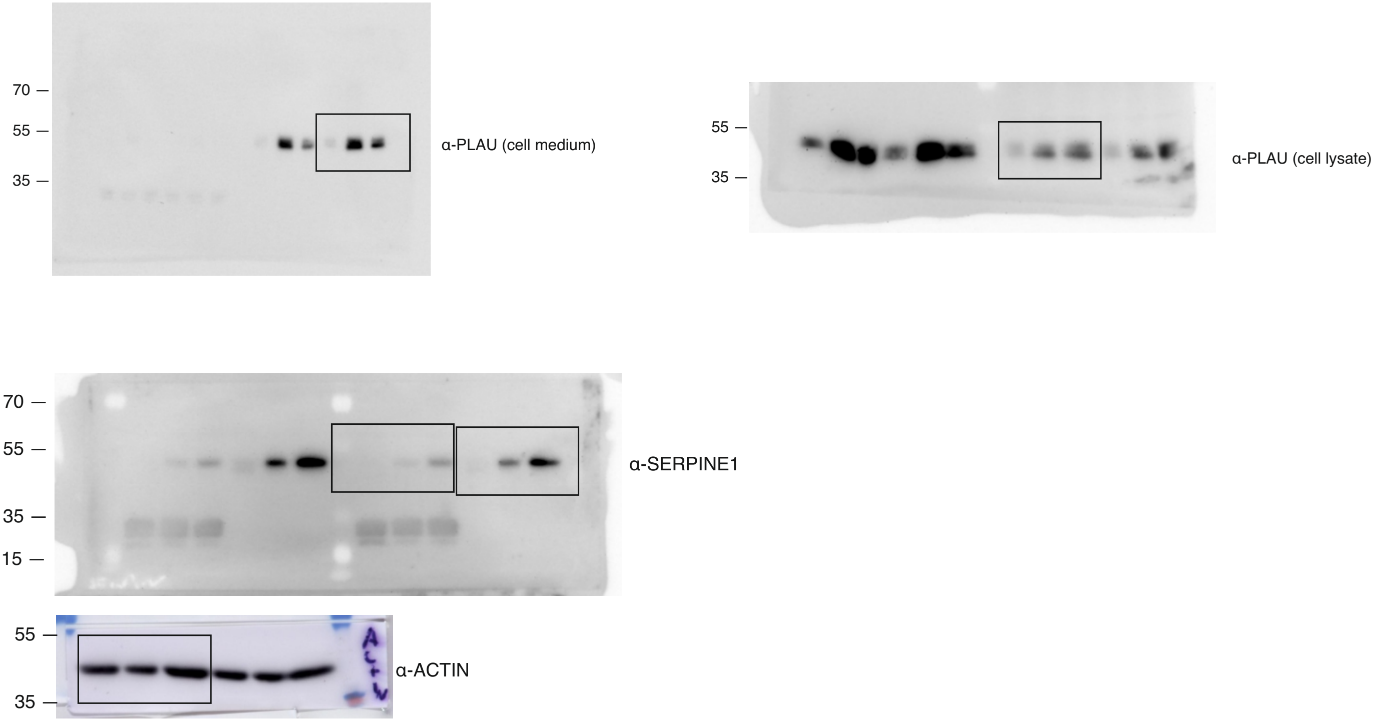

Supplementary Information 1. Uncropped images of the western blots analysis used for supplementary figure 1 and main figure 1. The black rectangles indicate the part that was kept for the final figure. When membrane edges are not visible, membranes were cut prior to hybridization with antibodies.

Fig. 2c

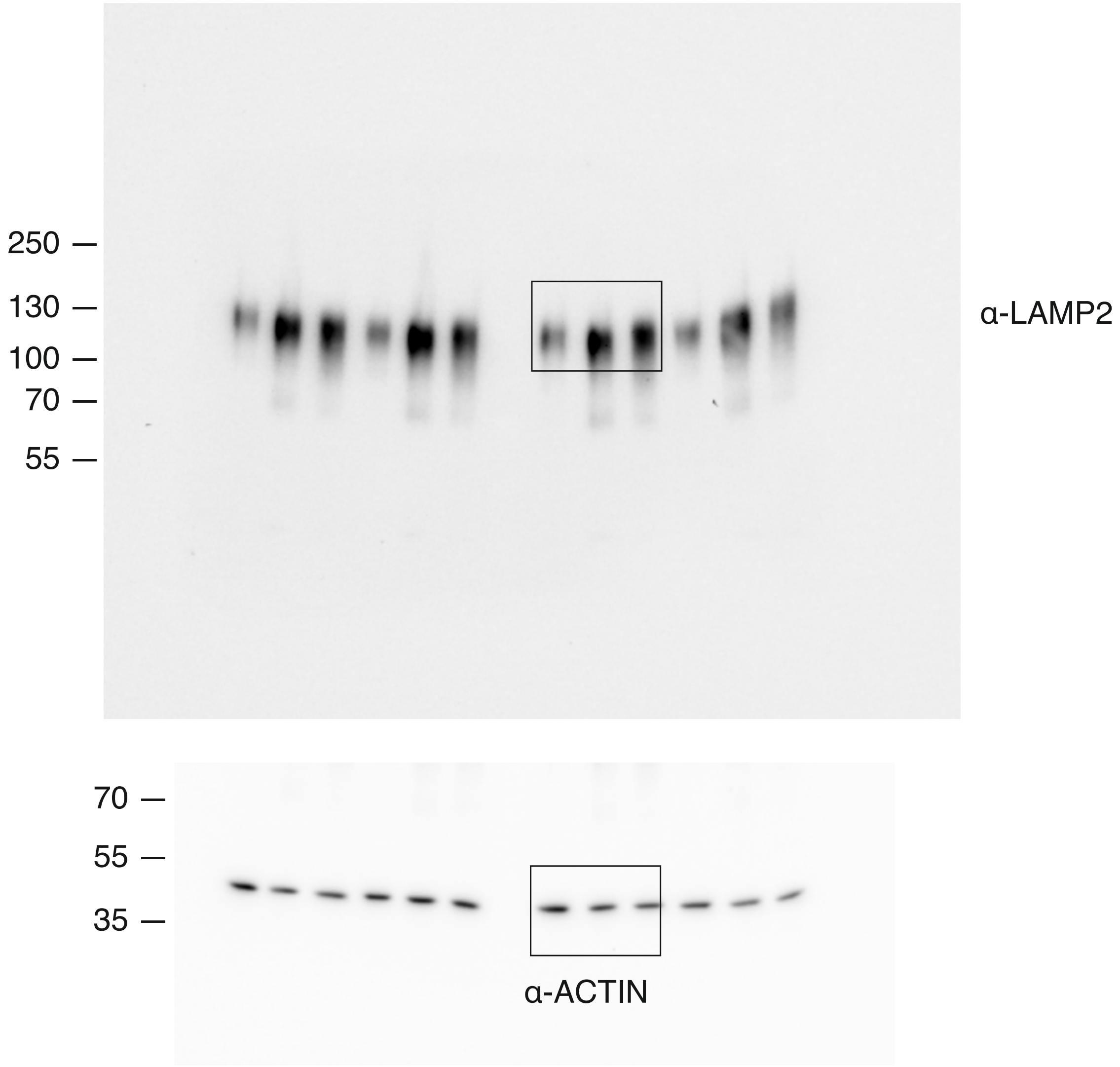

Fig. 2g

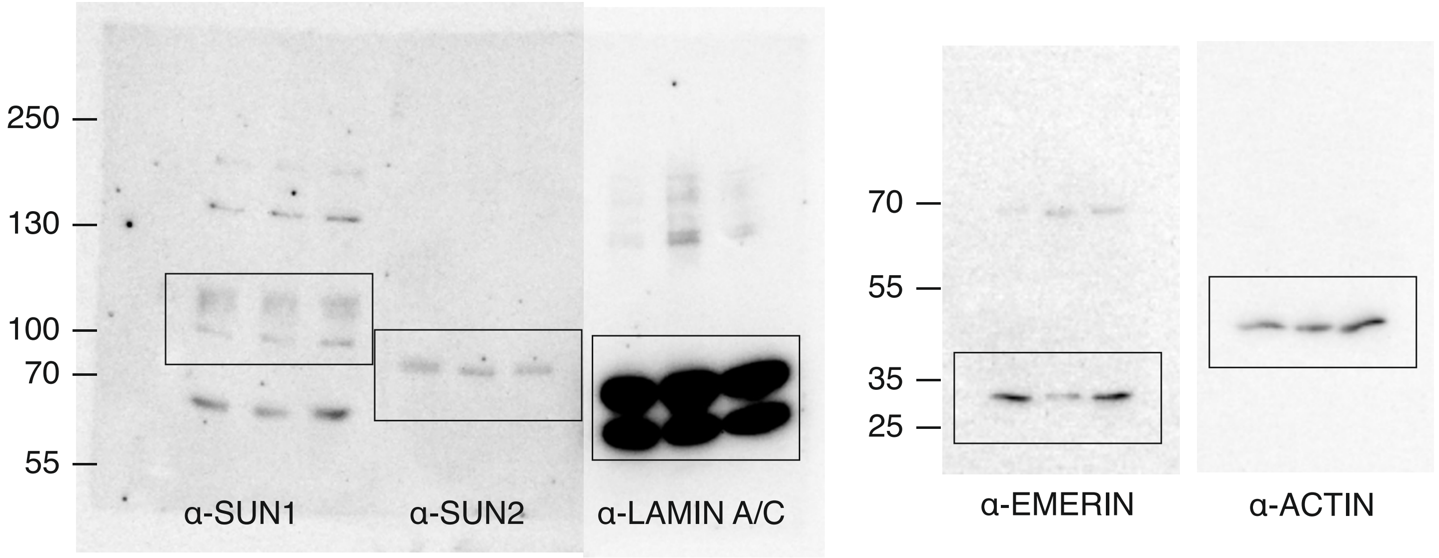

Supplementary Information 2. Uncropped images of the western blots analysis of the main figure 2. The molecular weights are indicated at the left of the immunoblots (in kDa). The black rectangle indicates the part that was kept for the final figure. When membrane edges are not visible, membranes were cut prior to hybridization with antibodies.

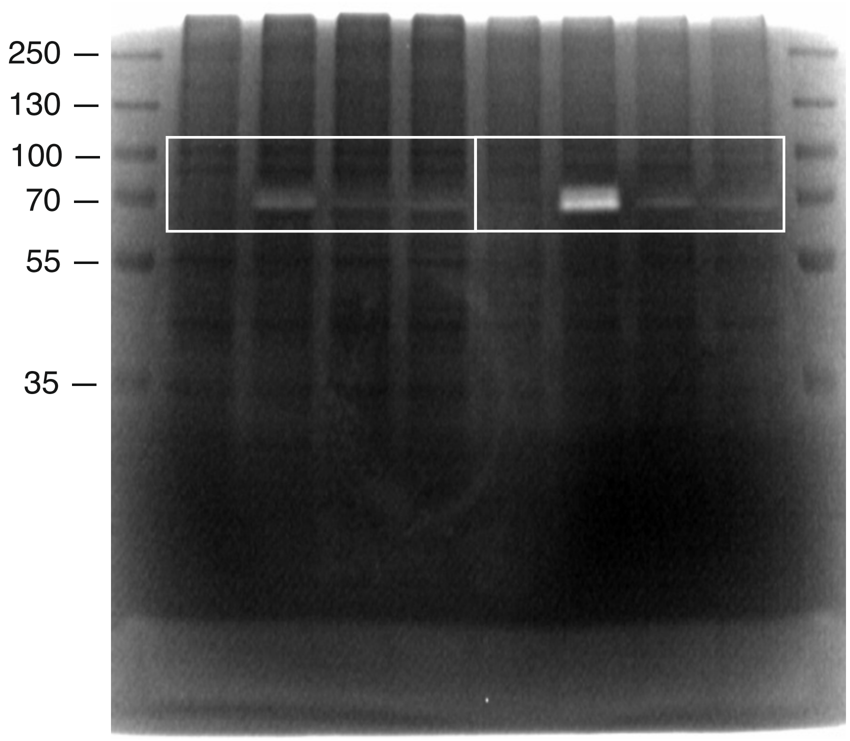

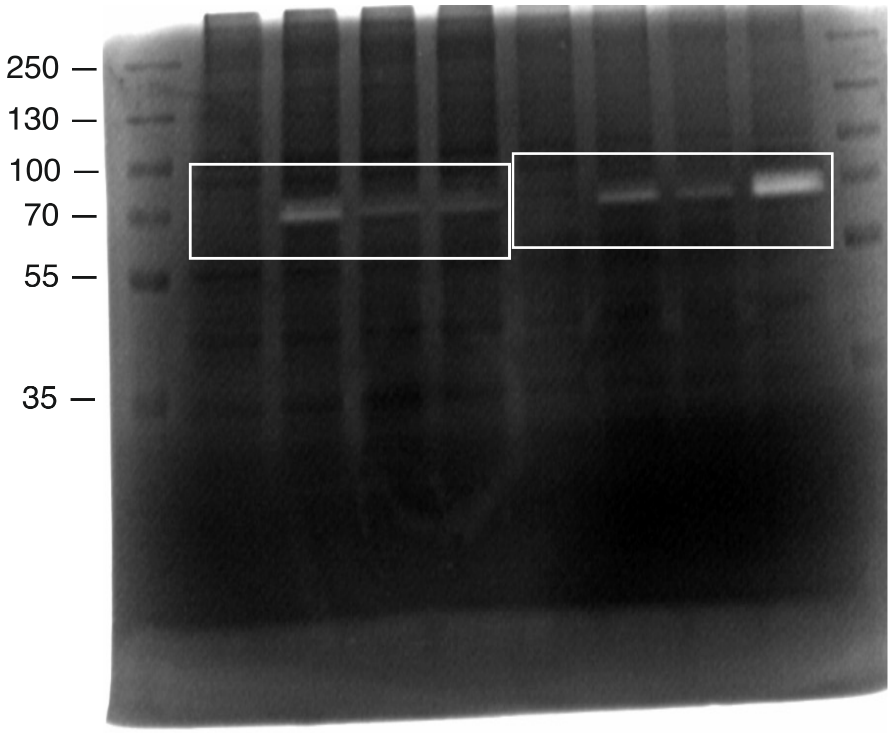

Supplementary Information 3. Uncropped images of the zymography analysis of the main figure 1. The molecular weights are indicated at the left of the gels (in kDa). The whiterectangle indicates the part that was kept for the final figure.

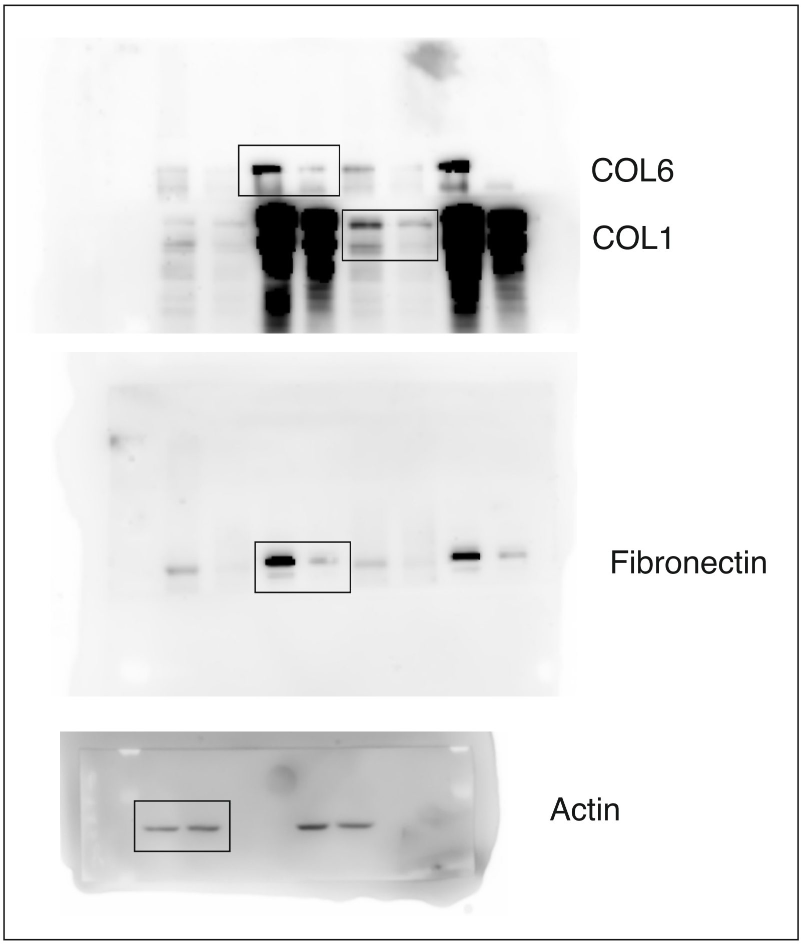

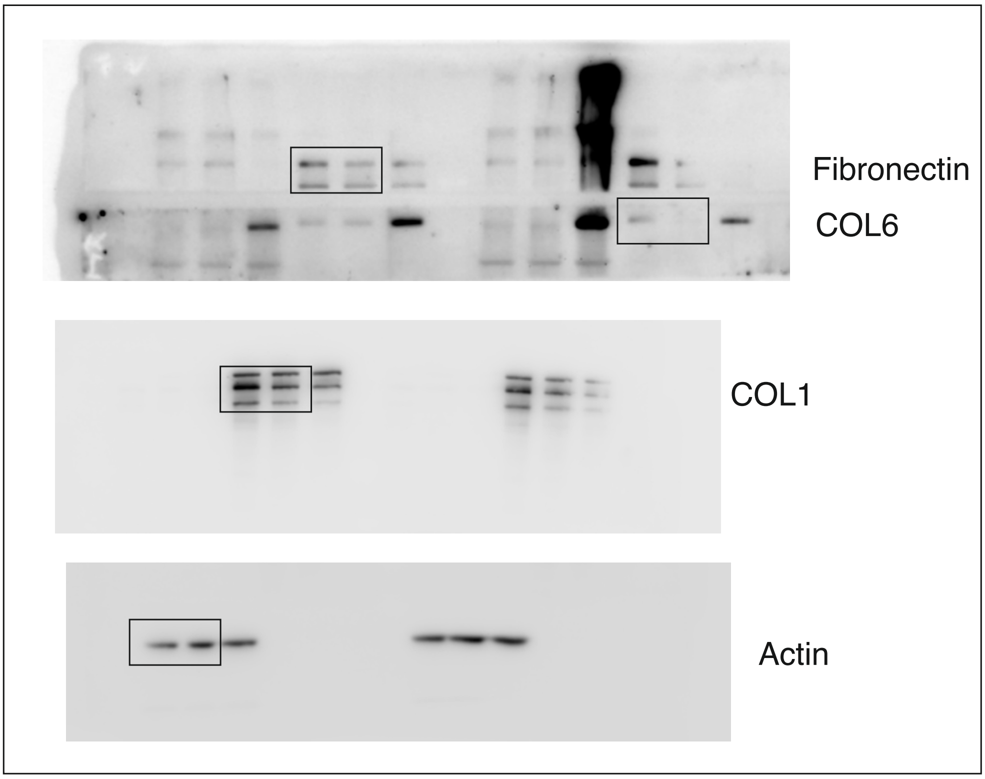

Supplementary Information 4. Uncropped images of the western blots analysis of the supplementary figure 4. The black rectangle indicates the part hat was kept for the final figure. When membrane edges are not visible, membranes were cut prior to hybridization with antibodies.

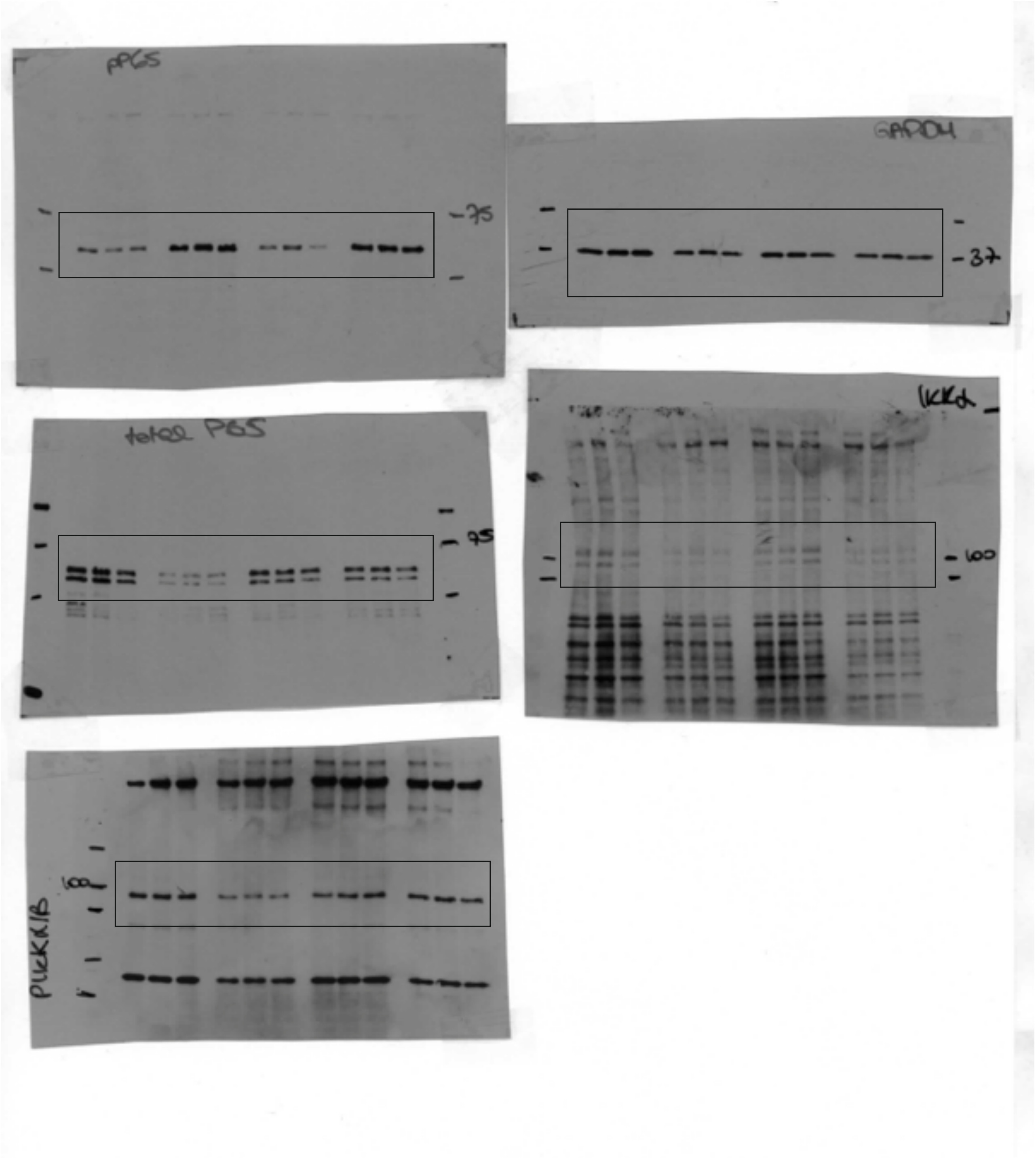

Supplementary Information 5. Uncropped images of the western blots analysis of the supplementary figure 6c. The black rectangle indicates the part hat was kept for the final figure. When membrane edges are not visible, membranes were cut prior to hybridization with antibodies.

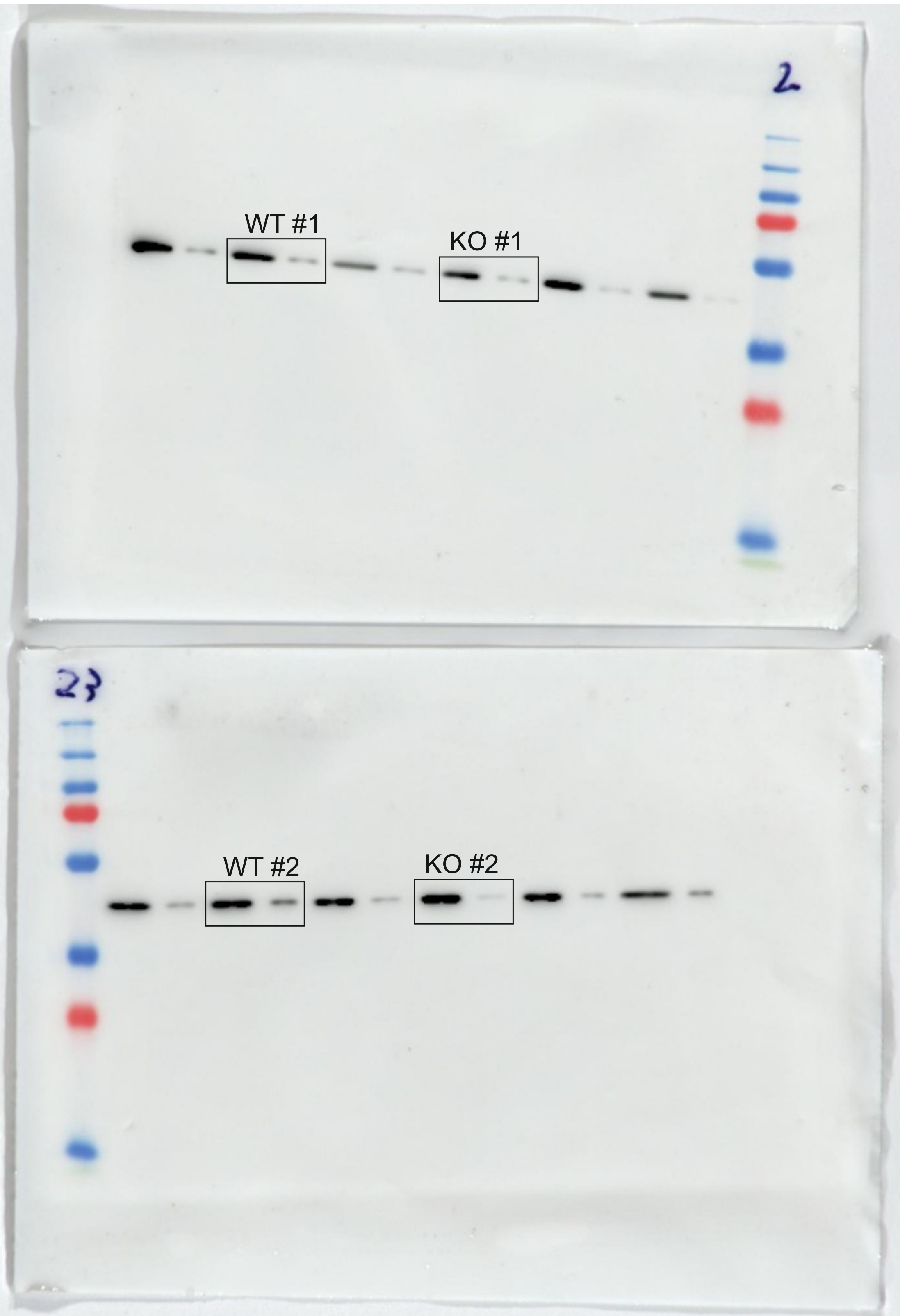

Supplementary Information 6. Uncropped images of the western blots analysis of the supplementary figure 8. The black rectangle indicates the part hat was kept for the final figure. When membrane edges are not visible, membranes were cut prior to hybridization with antibodies.
